# Mass Spectrometry-based Absolute Quantification of 20S Proteasome Status for Controlled *Ex-vivo* Expansion of Human Adipose-derived Mesenchymal Stromal/Stem Cells*[Fn FN2]

**DOI:** 10.1074/mcp.RA118.000958

**Published:** 2019-01-30

**Authors:** Thomas Menneteau, Bertrand Fabre, Luc Garrigues, Alexandre Stella, Dusan Zivkovic, Florence Roux-Dalvai, Emmanuelle Mouton-Barbosa, Mathilde Beau, Marie-Laure Renoud, François Amalric, Luc Sensébé, Anne Gonzalez-de-Peredo, Isabelle Ader, Odile Burlet-Schiltz, Marie-Pierre Bousquet

**Affiliations:** From the ‡Institut de Pharmacologie et de Biologie Structurale (IPBS), Université de Toulouse, CNRS UMR 5089, UPS, Toulouse, France;; §STROMALab, Université de Toulouse, INSERM U1031, EFS, INP-ENVT, UPS, Toulouse, France

**Keywords:** Absolute quantification, Selected reaction monitoring, Protein complex analysis, SILAC, Stem cells*, Targeted mass spectrometry, 20S Proteasome, Human Adipose-derived Mesenchymal Stromal/Stem Cells, stoichiometry

## Abstract

The proteasome controls a multitude of cellular processes through protein degradation and has been identified as a therapeutic target in oncology. However, our understanding of its function and the development of specific modulators are hampered by the lack of a straightforward method to determine the overall proteasome status in biological samples. Here, we present a method to determine the absolute quantity and stoichiometry of ubiquitous and tissue-specific human 20S proteasome subtypes based on a robust, absolute SILAC-based multiplexed LC-Selected Reaction Monitoring (SRM) quantitative mass spectrometry assay with high precision, accuracy, and sensitivity. The method was initially optimized and validated by comparison with a reference ELISA assay and by analyzing the dynamics of catalytic subunits in HeLa cells following IFNγ-treatment and in range of human tissues. It was then successfully applied to reveal IFNγ- and O_2_-dependent variations of proteasome status during primary culture of Adipose-derived-mesenchymal Stromal/Stem Cells (ADSCs). The results show the critical importance of controlling the culture conditions during cell expansion for future therapeutic use in humans. We hypothesize that a shift from the standard proteasome to the immunoproteasome could serve as a predictor of immunosuppressive and differentiation capacities of ADSCs and, consequently, that quality control should include proteasomal quantification in addition to examining other essential cell parameters. The method presented also provides a new powerful tool to conduct more individualized protocols in cancer or inflammatory diseases where selective inhibition of the immunoproteasome has been shown to reduce side effects.

## Abstract (precis)

20S proteasomes are very heterogeneous protein complexes involved in many cellular processes. In the present study, we combined an MRM-based assay with the production and purification of entire SILAC labelled proteasome to monitor absolute quantities of the different 20S proteasome subtypes in various human cells and tissues. This method applied to adipocyte-derived stem cells (ADSCs) amplified under various conditions highlights an increased expression of immunoproteasome when this type of cell is primed with IFNγ or amplified in a 20% O_2_ environment.

## Graphical Abstract

**Figure:**
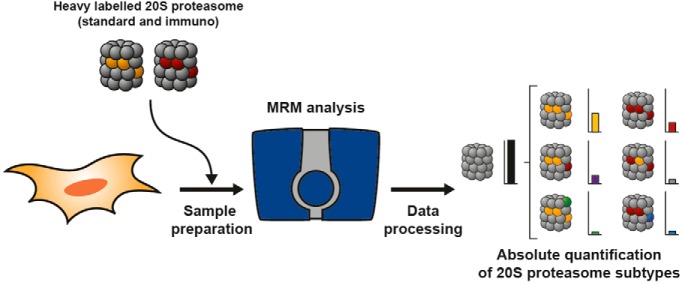

- Design of an MRM assay to determine the absolute quantity and stoichiometry of ubiquitous and tissue-specific human 20S proteasome subtypes.
- Use of purified isotopically labelled 20S proteasome as internal standard for accurate quantification.
- Variation in the expression of immunoproteasome in adipocyte-derived stem cells (ADSCs) grown under different O_2_ levels might be causal for change in cells differentiation capacity.
- The status of 20S proteasome during ADSCs expansion might constitute an additional relevant quality control parameter to contribute to predict, among other quality markers, their therapeutic capacity.

Some hematopoietic malignancies can be successfully treated by inhibition of the catalytic core 20S proteasome complex, and more recent findings indicate that the proteasome is a promising target for the treatment of other cancer types or other pathologies including inflammatory diseases (1–3).

Although the cylindrical α7β7β7α7 barrel-like structure of the 20S catalytic core proteasome has been preserved throughout evolution, the oligomeric protease has evolved, resulting in a higher heterogeneity of subunit compositions in mammals. As schematically represented in Fig. 1*A*, there exist at least six distinct forms of 20S proteasomes in human cells and tissues. The standard 20S proteasome (sP20S)^1^ is composed of constitutive (α1–α7 and β3, β4, β6, and β7) and catalytic subunits (β1, β2, and β5). It is the most abundant 20S subcomplex in most cell types, but significant amounts of other 20S forms have been observed in some human tissues and cells in their basal state or are induced in specific environmental conditions (4). For instance, in response to pro-inflammatory cytokines or in immune cells, the three catalytic subunits of the sP20S can be replaced in a highly regulated way by their immuno counterparts to form the immunoproteasome (iP20S), which has nonidentical cleavage specificities (5). Two intermediate proteasomes harboring a mixed assortment of standard and immunocatalytic subunits (β1i P20S, β1iβ5i P20S) have also been observed in various human tissues and cells, and their existence is consistent with the rules of cooperative assembly of inducible catalytic subunits (6). Some other 20S subtypes are much more tissue-specific, such as the thymoproteasome (containing β1i, β2i and β5t catalytic subunits) and the spermatoproteasome (containing a specific isoform of α4 called α4s) which are found in the thymus and male germ cells, respectively. The environment- or tissue-specific subunit composition of human 20S proteasome has been shown to fulfill specialized functions that the standard proteasome can only exert suboptimally (4). For example, immune cells contain a significant proportion of immunosubunit-containing 20S proteasome subtypes which display specific proteolytic preferences thanks to which they produce antigens for presentation to CD8 T cells (6). In the thymus, the unique cleavage preference of β5t explains the essential role played by the thymoproteasome in the positive selection of developing T lymphocytes (7). Thus, knowledge of 20S proteasome composition will help us to better understand proteasome function in a given tissue or cell type. It is also crucial when developing novel therapeutic strategies targeting specific 20S proteasome variants. Indeed, the immunoproteasome is a valuable target in several ongoing oncology trials as well as in the treatment of inflammatory and autoimmune diseases (2, 3, 8). Thus, to assess proteasome status, by determining the absolute quantity and stoichiometry of all subtypes, precise and accurate absolute quantification of several subunits from the same biological sample is required. As mRNA and protein levels reported from proteomic and transcriptomic analyses show a low correlation (9), quantification must be performed at the protein level to allow an accurate and complete description of the 20S proteasome status. Several ELISA protocols have been published or are commercially available to determine absolute levels of both 20S and 26S proteasome complexes, but only at the global level, *i.e.* they make no distinction between the different subcomplexes (6, 10–14). Only Guillaume *et al.* (6) considered the heterogeneity of 20S subtypes when developing their ELISA assay by using different in-house produced antibodies directed against four different standard and immunocatalytic β subunits. More recently, standard and immunoproteasome subtypes were determined by surface plasmon resonance imaging (SPRI) using specific inhibitors (15). However, the multiplexing capacity of these methods is insufficient to fully assess proteasome heterogeneity in a single assay.

To overcome this limitation, we propose to use Selected Reaction Monitoring (SRM), an isotope dilution mass spectrometry (IDMS)-based technique which has been approved for the quantification of multiple biomolecules. In addition to its robustness, precision and accuracy, the main advantage of SRM is its capacity to quantify many analytes simultaneously. SRM has recently been successfully applied in protein assays (16, 17). In these studies, the proteins themselves were not directly detected and quantified, but were analyzed at peptide level, after enzymatic proteolysis. This is a critical step because, in most cases, the absolute quantification relies on the addition of an isotopically-labeled peptide standard (called AQUA peptide) (18) late in the experimental workflow, which might introduce a high variability because of differences in sample preparation. Thus, for accurate and robust absolute quantification of proteins, and to determine protein stoichiometry within protein complexes, the use of isotope-labeled peptides as internal standards requires careful optimization of experimental conditions (19). An alternative approach based on artificial genes coding for concatenated proteotypic peptides (QconCAT strategy) (20) may be used to decrease quantification biases. For robust absolute quantification, an isotope-labeled equivalent of the full-length target protein is considered the ideal internal standard. Several approaches based on the synthesis of heavy versions of the protein of interest have emerged recently. Most of them rely on metabolic labeling and purification of a recombinant version of the protein of interest, or a shorter specific protein sequence, like the Protein Standards for Absolute Quantification (PSAQ) (21), absolute SILAC (22), PrEST (23), FlexiQuant (24), and TAQSI (25) methods. Another approach consisting in the chemical labeling of proteins using isobaric stable isotope tagging technology (26) has recently been described to reduce costs and the time required to grow cells in heavy amino acid-containing media. In all these approaches, because the internal standard is processed together with its endogenous analog throughout the whole workflow, accuracy is markedly better than achievable with AQUA and QconCAT approaches when determining absolute quantities of proteins in various matrices (17). However, to our knowledge, the absolute quantification relying on the isotopic dilution of labeled proteins has been limited so far to monomers, and no reports of assays to determine the stoichiometry of macromolecular complexes have been published.

Mesenchymal stem/stromal cells (MSC) hold great potential in regenerative medicine because of their multi/pluripotency and immunosuppressive properties. Over the last decade, the clinical use of MSC has rapidly increased, and more than 800 clinical trials assessing MSC therapy in multiple clinical settings are currently registered (https://www.clinicaltrials.gov/). Adipose-derived Stem Cells (ADSCs) are a subclass of mesenchymal stem/stromal cells initially derived from the bone marrow. However, to obtain the critical number of cells before transplantation, ADSCs must be expanded *in vitro*. This essential step has raised important concerns about the quality of adult stem cells, and interpatient variability is a challenge when seeking to define ADSCs. The development of uniform protocols for both preparation and characterization of MSCs, including standardized functional assays to assess their biological potential, will be critical in contributing to their clinical utility.

Here, we applied the SILAC method to engineered cell lines expressing either the standard 20S proteasome or the immunoproteasome to produce and purify isotope-labeled, endogenous versions of standard proteasome and immunoproteasome *in vivo*. These isotope-labeled proteasome complexes were used as internal standards to quantify the absolute concentration of all 20S catalytic and noncatalytic subunits in biological samples of various origins. The method developed was applied to determine the absolute concentration of total 20S proteasomes and the exact stoichiometry of six ubiquitous and tissue-specific 20S proteasome subtypes in a multiplexed LC-SRM assay with high precision (>92%), accuracy (>90%), and sensitivity (<1 fmol on column). Our results show that the absolute quantity and stoichiometry of the proteasome are challenged both by IFNγ stimulation and O_2_ levels during *ex vivo* expansion of primary ADSCs. Thus, determining proteasome status, which is a central contributor to maintaining stem cell homeostasis—characterized by stemness, capacity for self-renewal and cell differentiation (27–29)—might constitute an additional relevant quality control parameter for the production of ADSCs for clinical applications, which is of interest as the number of quality markers currently available is limited (30). Furthermore, accurate and precise assessment of proteasome abundance and heterogeneity could also help when seeking to achieve selective inhibition of a proteasome subtype, like the immunoproteasome, for personalized therapies in cancer or autoimmune diseases. This is the first study to report the simultaneous determination of absolute quantity and stoichiometry for macromolecular complexes based on the isotopic dilution of labeled proteins in numerous human tissues and primary ADSCs culture.

## EXPERIMENTAL PROCEDURES

### 

#### 

##### Cell Lines, Culture Conditions, SILAC, Human Samples

HEK 293T, HCT116, HeLa, and RKO cell lines were grown in DMEM medium supplemented with 10% fetal bovine serum (FBS). U937, HeLa S3, and NB4 cell lines were grown in RPMI 1640 medium supplemented with 10% FBS. KG1a cell line was grown in RPMI 1640 medium supplemented with 20% FBS. MRC5 cell line was grown in MEM-α medium supplemented with 10% FBS. All cultures were supplemented with 2 × 10^−3^ m glutamine, 100 units/ml penicillin, 100 μg/ml streptomycin, and maintained at 37 °C under 5% CO_2_. Unsynchronized cells were harvested at 80% confluence for adherent cells or at a concentration of 1×10^6^ cells per ml of culture for suspension cells. HeLa cells were treated with interferon-γ (R&D Systems, Minneapolis, MN) at 100 ng/ml in fresh medium.

Human 293-EBNA cells, “HEK-EBNA sP20S” (mainly expressing sP20S), and 293-EBNA cells engineered to express iP20S, “HEK-EBNA iP20S” (by transfecting 293-EBNA cells with cDNAs encoding the three immunocatalytic subunits β5i, β1i and β2i) were obtained as previously described (6). HEK-EBNA sP20S cells were cultured in SILAC medium which is composed of DMEM supplemented with 10% dialyzed FBS, 4 mm l-glutamine, 200 mg/L l-Proline, 100 mg/ml l-arginine (^13^C_6_), and l-lysine (^13^C_6_) (Cambridge Isotope Lab., Tewksbury, MA), 100 IU/ml penicillin and 100 μg/ml streptomycin in 150 cm^2^ culture plates and maintained at 37 °C under 5% CO_2_. HEK-EBNA iP20S were cultured in the same SILAC medium as HEK-EBNA sP20S, but further supplemented with 5 μg/ml Puromycin and 600 μg/ml Hygromycin to maintain selective pressure. Ten cellular doublings were performed in this medium to achieve an incorporation rate of 95% heavy amino acids in proteins (assessed by MS). Standard 20S proteasome and iP20S were then purified as described earlier (31). Absolute quantities and purities of both purified proteasome subtypes were then assessed as described in Supplementary information I-1. Isotope-labeled sP20S and iP20S were stored as 10-μl aliquots at 1.158 and 0.980 pmol/μl, respectively, in 20 mm Tris/HCl, pH 7.2, 1 mm EDTA, 1 mm DTT, 10% glycerol, and at −80 °C to ensure stability over time.

Whole-cell lysates from various human tissues were supplied by AMSBio (Cambridge, MA) (HT-201 (Brain); HT-311 (Colon); HT-804 (Heart); HT-314 (Liver); HT-601 (Lung); HT-406 (Ovary); HT-102 (Skeletal muscles); HT-701 (Spleen); HT-401 (Testis); HT-704 (Bone Marrow); P1234264 (Thymus)).

Human ADSC were isolated from subcutaneous adipose tissue (AT) obtained from nonobese human donors (body mass index <26) undergoing elective abdominal dermolipectomy (Plastic Surgery Department, Rangueil Hospital, Toulouse, France). No-objection certificates were obtained to comply with bioethics law no. 2004–800 of 6 August 2004. The stromal vascular fraction (SVF) was obtained by enzymatic digestion of adipose tissue (AT) with collagenase NB4 (Roche Diagnostics, Indianapolis, IN). Cells were then seeded at 4000 cell/cm^2^ (P0) and cultivated in α-minimal essential medium supplemented with 10% FBS (Gibco), 100 μg/ml streptomycin, 100 U/ml penicillin, 25 μg/ml amphotericin (Thermo Fisher Scientific Life Sciences, Waltham, MA). Medium was changed twice a week. Cells from the same patients were cultured under normoxic (20% O_2_, 5% CO_2_) or hypoxic (1% or 5% O_2_, 5% CO_2_) conditions in an Xvivo System (BioSpherix, Paris, NY) to maintain cells in hypoxic conditions at all culture steps. Interferon-γ (100 ng/ml) was added to the medium in some experiments.

Our experimental protocols were approved by French research ministry's institutional ethics committee of (N°: DC-2015-23-49) and informed consent was obtained from all subjects in line with current regulations (no subjects age under 18 were included).

##### Adipocyte Differentiation

ADSCs obtained from patients were seeded at 20,000 cell/cm^2^ and were exposed to an adipogenic mixture containing IBMX (3-isobutyl-1-methylxanthine) 0.45 mm, Dexamethazone 1 μm and Indomethacine 60 μm. An immunoproteasome inhibitor, 100 nm ONX-0914 (solubilized in 0.001% DMSO), was also added to the cell medium in some conditions; 0.001% DMSO was added to the control condition in these cases. Cells were grown at 37 °C under normoxic (20% O_2_) or hypoxic conditions (5% and or 1% O_2_). The medium was changed every 2–3 days throughout the culture process. At the end of experiment, the cell lineage was determined using Oil Red O which stains for adipocytes.

##### 20S Proteasome Purification

When they reached 80% confluence, HEK-EBNA cell lines (HEK-EBNA sP20S or HEK-EBNA iP20S) were harvested in HKMG buffer (10 mm Hepes pH 7.9, 10 mm KCl, 5 mm MgCl_2_, 10% glycerol, 10 mm ATP, 1% NP40, protease and phosphatase inhibitor (Roche, Bâle, Switzerland)) and centrifuged for 10 min at 10,000 × *g*. Supernatants were kept and used for proteasome immuno-purification as previously described (31).

##### ELISA-based Absolute Quantification of the 20S Proteasome

ELISA-based absolute quantification of the 20S proteasome was performed as previously described (14). Briefly, ELISA assays were performed in 96-well plates (IMMULON HBX 4, Thermo Scientific). The plate was coated with 100 μl MCP21 monoclonal antibody (European Collection of Cell Cultures) at 5 μg/ml by incubation at 4 °C overnight. Wells were then washed 3 times with PBS/T-buffer. Nonspecific sites were blocked by incubation with 2% BSA in PBS for 1 h at room temperature with slow shaking. The plate was washed 3 times with PBS/T-buffer. The samples (cell lysates) were then deposited in triplicate and incubated for 2 h at room temperature. The plate was washed with PBS/T-buffer and then incubated with the polyclonal rabbit anti-20S antibody (PW 8155, ENZO LIFE Sciences, Farmingdale, NY) for 1 h at room temperature under slow shaking. The plate was washed 3 times with PBS/T-buffer and antibody binding was revealed using horseradish peroxidase-conjugated anti-rabbit antibody and 2 mg/ml ABTS substrate (2,2-azino-bis(3-ethylbenzthiazoline-6-sulfonic acid)). The reaction was monitored by measuring the optical density at 416 nm (μQuant; Bio-Tek instruments, Inc., Winooski, VT). The amount of proteasome in the sample was calculated by comparison with the calibration curve produced with purified 20S proteasome purified from human erythrocytes (ENZO LIFE SCIENCE). A linear dose-response was observed between 0 and 20 ng.

##### Proteasome Activity Assay

Cultured cells were harvested in HKMG buffer and sonicated in ice with a Bioruptor Plus (Diagenode, Liège, Belgium) (15 min, cycle 45 s/15 s (ON/OFF), position High). Protein concentration was determined by detergent-compatible protein assay (DC Assay - BioRad, Hercules, CA) according to the manufacturer's recommendation. Proteasome activity was assayed in 96-well black plates (Greiner Bio-One, Frickenhausen, Germany). 10 μl of each lysate fraction were added to 40 μl of Tris-HCl 100 mm and 50 μl of Suc-LLVY-AMC (for chymotrypsin-like activity), Boc-LRR-AMC (for trypsin-like activity) and Z-LLE-AMC (for PGPH activity) substrate in 200 mm Tris-HCl, pH 8 (Enzo Life Science) at a final concentration of 400 μm/well. Kinetic assays were performed at 37 °C in a FLX-800 spectrofluorometer (BIOTEK) over 90 min, reading fluorescence every 5 min, at 460 nm following excitation at 360 nm.

##### Samples Preparation and In-gel Digestion for Mass Spectrometry Analysis

Samples were heated to 95 °C for 5 min in Laemmli buffer to denature proteins; 100 mm chloroacetamide was then added to the sample followed by incubation for 30 min at room temperature in the dark. Proteins were loaded onto a 12% acrylamide SDS-PAGE and concentrated in a single band, visualized by Coomassie staining (Instant Blue - Expedeon). The gel band containing the whole sample was cut and washed several times in 50 mm ammonium bicarbonate, acetonitrile (1:1) for 15 min at 37 °C. Trypsin (Promega) digestion was performed over night at 37 °C in 50 mm ammonium bicarbonate at a trypsin/total protein ratio of 1/50. Peptides were extracted from the gel by two incubations in 10% formic acid, acetonitrile (1:1) for 15 min at 37 °C. Extracts were dried in a SpeedVac, and resuspended in 2% acetonitrile, 0.05% trifluoroacetic acid prior to LC-MS/MS analysis.

For LC-SRM analysis, 1 pmol of labeled iP20S and 1 pmol of labeled sP20S were added to 25 μg of total proteins before the protein denaturation step. When used, AQUA peptides (Thermo Scientific Pierce Protein Research) were spiked into samples just before mass spectrometry analysis at a concentration resulting in injection of a final quantity of 70 fmol.

##### Whole Proteome Analysis of Extracts from Human Tissues and Adscs Grown Under Normoxic and in Hypoxic Conditions (1% O_2_)

Peptide mixtures were analyzed by nano-LC-MS/MS using an Ultimate3000 system (Dionex Sunnyvale, CA) coupled to an LTQ-Orbitrap Velos mass spectrometer (Thermo Fisher Scientific) when analyzing human tissue extracts or to a Q-Exactive Plus mass spectrometer (Thermo Fisher Scientific) when analyzing ADSCs. Five microliters (human tissue) or two microliters (ADSCs) of each peptide sample at 1 μg/μl were loaded onto a C18 precolumn (300 μm inner diameter × 5 mm; Thermo Scientific) at 20 μl/min in 5% acetonitrile, 0.05% trifluoroacetic acid. After 5 min of desalting, the precolumn was switched online with the analytical C18 column (75 μm inner diameter x 50 cm; home-made) equilibrated in 95% solvent A (5% acetonitrile, 0.2% formic acid) and 5% solvent B (80% acetonitrile, 0.2% formic acid). Peptides from human tissues were eluted using a 5–50% gradient of solvent B over 105 min at a flow-rate of 300 nL/min and peptides from ADSCs were eluted using a 5–25% gradient of solvent B over 165 min and a 25–50% gradient of solvent B over 135 min at a 300 nl/min flow rate. Both mass spectrometers were operated in data-dependent acquisition mode. During the analysis with the LTQ-Orbitrap Velos, survey scan MS spectra were acquired in the Orbitrap over the 350–1800 *m*/*z* range with resolution set to 60,000 (these parameters were 350–1500 *m*/*z* and 70,000 with Q-Exactive Plus, respectively). On the LTQ-Orbitrap Velos, the twenty most intense ions per survey scan were selected for CID fragmentation, and the resulting fragments were analyzed in the linear trap (LTQ). On the Q-Exactive Plus, the ten most intense ions per survey scan were selected for HCD fragmentation, and the resulting fragments were analyzed in the Orbitrap. Dynamic exclusion was used with a 60-s or a 30-s window (on the LTQ-Orbitrap Velos or the Q-Exactive Plus, respectively) to prevent repeated selection of peptides.

Raw mass spectrometry files were processed using MaxQuant (version 1.5.5.1) and Andromeda was used to match MS/MS spectra against the Human SwissProt database (March 2017 release - 20,181 entries for human tissues analysis/release of August 2018 - 20,386 entries for ADSCs analysis) and a list of potential contaminant sequences provided in MaxQuant1.5.5.1., with Carbamidomethylation of cysteines set as fixed modification. Oxidation of methionine and protein N-terminal acetylation were set as variable modifications. The digestion specificity of trypsin was defined as cleavage after K or R, and up to two missed trypsin cleavage sites were allowed. The precursor mass tolerance was set to 20 ppm for the first search and 4.5 ppm for the main search. The mass tolerance in MS/MS mode was set to 0.8 Da for tissue analysis and to 20 ppm for ADSC analysis. Minimum peptide length was set to seven amino acids, and minimum number of unique peptides was set to 1. A target-decoy approach was used to validate hits, using a reverse database and applying a peptide and protein false-discovery rate of 1%. The “match between runs” option in MaxQuant was enabled, with a time window of 0.7 min, to allow cross-assignment of MS features detected in different runs. Only unique peptides were used for quantification when analyzing human tissues, whereas both unique and razor peptides were used for the quantification when dealing with ADSCs data. Quantitative proteomic analysis was performed on the normalized LFQ intensities from the “proteinGroups” Table in the MaxQuant output. Protein entries identified as potential contaminants by MaxQuant were eliminated from the analysis, as were proteins identified by fewer than two peptides. Tissue-specific 20S proteasome subunits (α4s, β1i, β2i, β5i, and β5t) were compared across the different tissues based on their normalized LFQ intensities (Fig. 3*B*). To compare the proteomes of ADSCs grown under normoxic or hypoxic conditions, the LFQ intensity values were used for the quantitative analysis. Fold-changes were log_2_ transformed and thresholds calculated based on their distribution. The upper threshold was calculated as Q3 + 1.5 × IQ, and the lower threshold as Q1 − 1.5×IQ, where IQ is the interquartile, and Q1 and Q3 are the first quartile and the third quartile, respectively. Proteins with fold-changes outside these thresholds were considered outliers from the global distribution. Outlier proteins with a *p* value of less than 0.05 were considered as differentially expressed between normoxic and hypoxic conditions. GO terms enrichment analysis was performed on these proteins using GOrilla (32).

##### Multiple Reaction Monitoring to Quantify 20S Proteasome Subunits

Dried peptide samples were resuspended in a solution containing 2% acetonitrile, 0.05% TFA, to obtain a final concentration of 1 μg/μl. To obtain data for the whole set of peptides/transitions, samples were injected twice as the list of transitions was split into two methods. The sample (2.5 μl, about 2.5 μg protein equivalent) was loaded onto the system and analyzed on a hybrid triple quadrupole-ion trap mass spectrometer 6500 QTrap (AB Sciex, Framingham, MA) equipped with a nanoelectrospray ion source coupled to an Ultimate 3000 system (Dionex) for chromatographic peptide separation. Separation was achieved using a 60 min gradient from 0 to 50% of solvent B (80% acetonitrile, 0.2% formic acid) at a flow-rate of 300 nL/min. Spray voltage was set to 2500 V, curtain gas to 35 psi, nebulizer gas to 5 psi, interface heater temperature to 75 °C, and cycle time to ∼3 s (3.1620 s and 3.0380 s for the two methods). Peptides were loaded onto a C18 precolumn (300 μm inner diameter × 5 mm; Thermo Scientific) at 20 μl/min in 2% acetonitrile, 0.05% trifluoroacetic acid. After 5 min of desalting, the precolumn was switched online with the analytical C-18 column (75 μm inner diameter × 50 cm; in-house-packed with Reprosil C18) and equilibrated in solvent A (5% acetonitrile, 0.2% formic acid).

Proteotypic peptide sequences were selected based on results of previous discovery experiments (33–35) and by referring to the golden rules (16) (*i.e.* considering isoforms, variants, PTMs reported on protein sequence or possibly artifactually-induced by sample handling, or missed cleavages, observed in Protein (UniProt) or MS databases (PeptideAtlas)). To achieve maximal sensitivity, collision energies (CEs) were optimized within a 6 V window around the CE value recommended by Skyline software and based on the *m*/*z* of the precursor. Final SRM transitions are given in supplemental Data S3. Transitions could be unambiguously assigned thanks to the co-injected isotope-labeled peptides. Samples were run in a blinded fashion except for calibration curves, for which the lowest concentrations were injected first. To check system suitability and performance before injecting each batch of samples, Total Ion Current was tuned (chromatographic solvent ions of *m*/*z* 50 to 1000) at 2000 Da/s scan speed in Q1. Quality controls (QC) (20 fmol of tryptic digest of betagalactosidase; transitions are given in supplemental Data S4) were injected between each sample. Carry-over was checked by injecting tryptic digests of 100 fmol, 500 fmol or 1000 fmol of isotopically labeled sP20S and iP20S spiked in 2.5 μg of HeLa, then one QC sample, and finally a blank sample (the maximum carry-over observed was less than 0.01%). Quantitative reproducibility over time was checked based on signal intensities for heavy transitions (maximum deviation of 50% allowed); injection batches were generally carried out over periods of less than 1 week.

Quantitative data analyses were performed using Skyline-Daily open-source software (36). Area values for all transitions were first extracted automatically by the software, then checked manually and adjusted if necessary (*i.e.* exclusion of data points if S/N < 10). Light transition (L) peak area signals were then normalized with respect to their labeled counterparts (H) (after correction by considering the 95% incorporation rate for R(^13^C_6_) and K(^13^C_6_) in labeled sP20S and iP20S). For each transition, technical replicates of injection (typically three) were averaged. Ratios (L/H) of all the transitions used to assay a given protein or for total 20S proteasome (details in supplemental Data S3) were then averaged. The amount of labeled reference mix (containing equimolar concentrations of isotope-labeled sP20S and iP20S) spiked into the biological sample before sample preparation was then used to determine the absolute quantity of each proteasome subunit or total 20S proteasome. For each transition, LOD and LLOQ were experimentally determined by injecting heavy-isotope-labeled sP20S and iP20S spiked at increasing concentrations in a HeLa protein lysate and processed by the same method as applied to the other biological samples. LOD and LLOQ values were calculated using QuaSAR (37), which was implemented through the Skyline interface (see supplemental Data S5).

Targeted MS measurements were highly multiplexed, and used to quantify proteasome subunits across cell lysates, tissues and primary cells of human origin. The assay developed used internal standards for each analyte, to confidently detect and precisely quantify the proteins of interest. Thus, the analyses meet the expectations of Tier 2 level.

##### Determining the Stoichiometry of the Six Major 20S Proteasome Subtypes

The concentrations and stoichiometries of all proteasome subtypes were determined as explained hereafter.

Total proteasome absolute quantity was calculated by averaging the quantities of all 20S noncatalytic subunits (α1–7; β3, β4, β6, β7) (mean (α1,2,3,5,6,7; β3,4,6,7) → Total P20S). A stoichiometry of two noncatalytic subunits per 20S proteasome and a molecular weight of 700,000 g/mol were used in these calculations.

As β5 is integrated into the standard proteasome, its fraction exclusively represents the fraction of standard proteasome (β5 → sP20S). Similarly, the level of β5t corresponds to the fraction of thymoproteasome “β5t P20S”, and can be obtained by subtracting the sum of β5 and β5i levels from the total amount of 20S proteasome (Total P20S − β5 − β5i = β5t → β5t P20S). As the β2i subunit is integrated into both the immunoproteasome and the thymoproteasome, the difference in its quantity compared with β5t can be used to determine the fraction of immunoproteasome (β2i − β5t → iP20S). The β1i-containing proteasome subtypes correspond to immunoproteasome, thymoproteasome, and intermediate proteasome β1i− β5i. Thus, the quantity of β1i β5i P20S is equal to the difference in abundance of β1i and β2i proteins because both immunoproteasome and thymoproteasome contain β2i (β1i − β2i → β1iβ5i P20S). β5i is contained in the immunoproteasome and in both types of intermediate proteasomes (β5i **→** iP20S + β5i P20S + β1iβ5i P20S). Thus, the amount of β5i P20S can be calculated as follows: β5i + β5t − β1i = Total P20S − β1i − β5 → β5i P20S.

Finally, the spermatoproteasome level can be determined from the quantity of the α4s isoform (PSA7L protein _PSMA8 gene) by subtracting the abundance of the α4 subunit from the total 20S proteasome content (using specific peptides of the PSA7 isoform_PSMA7 gene as detailed in supplemental Fig. 8*A*) (sP20S − α4 → α4s P20S).

Thus, the absolute amounts of noncatalytic subunits, as well as those of the catalytic β2i, β5, β5i and β1i subunits determined by the SRM method were used to calculate the proportions of the six main 20S proteasome subtypes, as summarized below:

mean (α1,2,3,5,6,7; β3,4,6,7) → Total P20S
β5 → sP20S
Total P20S − β5 − β5i = β5t→ β5t P20S
β2i − β5t → iP20S
β1i − β2i → β1iβ5i P20S
P20S − β1i − β5 → β5i P20S
sP20S − α4 → α4s P20S

##### Experimental Design and Statistical Rationale

##### SRM Analyses

All statistical analyses were performed on at least three independent biological replicates. For each biological replicate, results from at least two injection replicates were *averaged. Probability values (p*) were determined by one-way and two-tailed analysis of variances (ANOVA). Differences were statistically significant at confidence levels of 95% (*), 99% (**), or 99.9% (***).

Coefficients of variation were calculated as ratios of the standard deviation over the mean of the values and are expressed as a percentage. Accuracy was determined as the ratio of the difference of the experimental value and the reference value over the reference value and is expressed as a percentage.

##### Label-free MS Analyses (Fig. 3B and supplemental Fig. 11)

The “human tissue extract” data set contains mass spectrometry results from the analysis of 11 different human tissues analyzed in triplicate, corresponding to 33 raw files. The “ADSC” data set contains mass spectrometry results from the analysis of three different patients under two different oxygen percentages used for cell culture, samples were analyzed in triplicates, and thus produced 18 raw files. Quantitative proteomic analysis was performed on the normalized LFQ intensities from the “proteinGroups” Table in the MaxQuant output.

The data sets corresponding to the mass spectrometry analyses presented in this study have been deposited in the following repositories: PRIDE (Project accession: PXD011894) for label-free MS analyses, and PeptideAtlas (Dataset identifier: PASS01219) for targeted MS analyses. The detailed descriptions of all analyses (raw and processed file names, sample name, biological replicate number, MS technical replicate number, corresponding figure) are summarized in supplemental Data S8.

## RESULTS

### 

#### 

##### Design and Validation of the SRM Assay to Determine 20S Proteasome Status

**Fig. 1.**
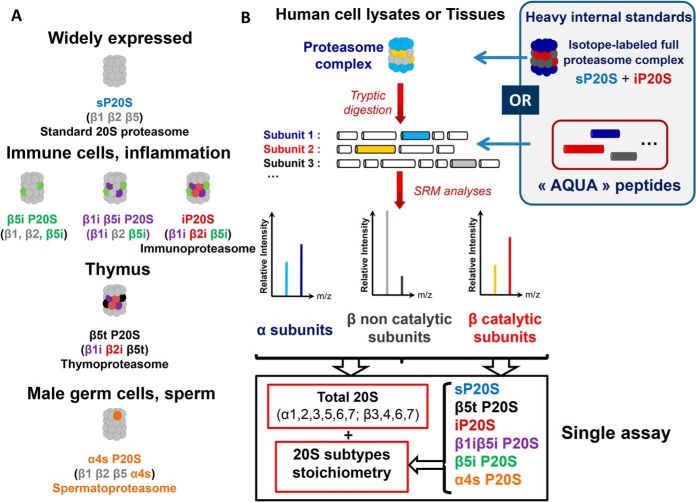
**Workflow for determination of total 20S proteasome absolute quantity and stoichiometry by LC-SRM.** *A*, Schematic representation of the six main 20S proteasome subtypes with different sets of subunits. The sP20S is the most abundant 20S subtypes in most cell types. The 20S proteasome subtypes containing at least one immunocatalytic subunit are predominantly observed in immune cells but can also be induced in response to inflammatory cytokines. Other 20S proteasome subtypes are tissue-specific, like the thymoproteasome (β5t P20S) and the α4s proteasome (α4s P20S) which have been observed in the thymus and in reproductive cells like testes, respectively. *B*, Determination of total 20S proteasome quantity and 20S subtypes stoichiometry in Human cell lysates or tissues using SRM. Endogeneous proteasomes contained in biological samples were digested with trypsin. The SRM approach was designed to quantify in a multiplexed manner a set of proteotypic peptides corresponding to α subunits, as well as β catalytic and noncatalytic subunits. Isotope-labeled standard and immunoproteasome complexes were used as internal standards in the final method. They were spiked in the biological sample at equimolar quantities before the tryptic digestion. Alternatively, heavy “AQUA” peptides were added to the endogeneous peptides lysate after tryptic digestion. At least three endogeneous peptides, and three transitions per peptide were analyzed. Thanks to internal normalization with the isotopically-labeled peptides transitions, the absolute quantity of selected α and β noncatalytic subunits as well as of β catalytic subunits were obtained and used to compute the absolute quantities of total 20S proteasome and to assess the stoichiometries of the six main 20S proteasome subtypes in biological samples. Details are provided in the Experimental Procedures section and in Experimental section.

**Fig. 2.**
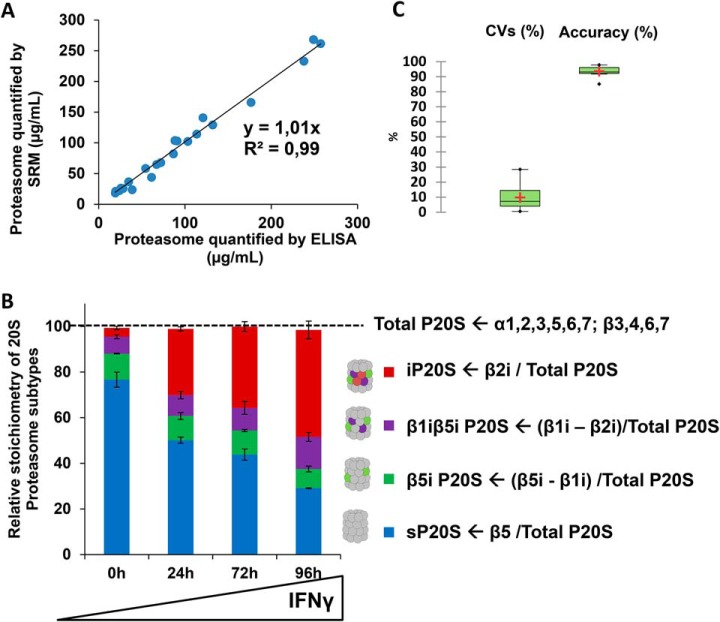
**Final validation of the LC-SRM method to determine absolute quantity of total 20S proteasome and to precisely and accurately monitor the dynamics of 20S subtypes stoichiometries.** *A*, Correlation curve between the 20S proteasome concentration measured by the ELISA reference method and the one determined by LC-SRM in a panel of eight human cell lines (HEK 293T, HCT116, RKO, U937, HeLa S3, NB4, KG1a, and MRC5). The LC-SRM method was based on the isotopic dilution of equimolar amounts of in-house isotopically-labeled sP20S and iP20S (R(^13^C_6_) and K(^13^C_6_)) in each cell line protein lysate. One peptide sequence (and three transitions for the light and the heavy surrogate) and three peptide sequences were analyzed for noncatalytic subunit (α1–7; β3,4,6,7) and catalytic subunits (β1,2,5,1i, 2i, 5i), respectively (see supplemental Data S3 for more details on peptides and transitions). An equivalent of 2.5 μg total proteins containing the sP20S and iP20S heavy internal standard mix were injected on column and analyzed by SRM, as detailed in the Experimental Procedures. Three biological replicates (and three technical replicates) were analyzed to obtain statistics. The total proteasome concentrations measured in the initial protein lysates are presented. *B*, Dynamics of 20S subtypes stoichiometries upon IFNγ stimulation of HeLa cells. The absolute quantities of each of the six catalytic subunits measured by the LC-SRM method were computed to calculate the stoichiometries of 20S proteasome subtypes as detailed under Experimental Procedures. HeLa cells were stimulated for 0, 24, 72, or 96 h with IFNγ and the SRM method used to quantify proteasome subunits in each total cell lysate obtained at each time point of cytokine stimulation. Peptides sequences, transitions and applied voltages are detailed in supplemental Data S3. Three biological replicates were analyzed to obtain statistics. The sum of the stoichiometries of the four 20S proteasome subtypes is very close to the expected value of 100 (obtained from independent measurement of total 20S proteasome absolute quantity). *C*, Method variability (CV %) and accuracy (%) calculated with data obtained from IFNγ-stimulated HeLa cells. Accuracy was calculated from comparison of total 20S proteasome quantity obtained from noncatalytic subunits (reference values) and from β1/β1i, β2/β2i, and β5/β5i couples of catalytic subunits (experimental values) (*n* = 36). Accuracy were computed as follow: accuracy = (experimental value − reference value)/reference value × 100. Method variability: Coefficients of variation on all independent measurements (*n* = 24). Details on stoichiometry determination of 20S proteasome subtypes are provided under Experimental Procedures.

The method aimed to simultaneously determine the absolute 20S proteasome quantity and subtype stoichiometry in a single assay. To achieve this goal, we designed a workflow combining IDMS and SRM to exploit its multiplexing capacity, sensitivity and robustness. In terms of total 20S proteasome absolute quantification, preliminary optimizations were used to select the most appropriate mode of internal standardization to correct for a range of experimental biases (Fig. 1*B*; supplementary Information I-1). As detailed in supplementary Information I-1 and supplemental Figs. S1–S5, the isotope-labeled whole proteasome complex added to biological preparations clearly provide a much more robust absolute quantification method than spiking with individual isotope-labeled “AQUA” proteotypic peptides for each 20S subunit. Indeed, despite good analytical performance in terms of reproducibility and linearity, absolute quantification using AQUA peptides technology lacked accuracy. This critical issue was overcome by using the absolute SILAC quantification method. We produced and affinity-purified isotope-labeled standard proteasome and immunoproteasome to obtain highly pure standards with a high level of subtype purity (min 99%) and with an excellent isotopic incorporation rate (min 95%) (supplementary Information I-1, supplemental Figs. S3–S4). The optimized quantification method then consisted in the distribution of a reference mixture of 20S proteasome subunits with carefully-controlled stoichiometry into protein lysates (Fig. 1*B*). After digestion of proteins using trypsin, an SRM method including at least one peptide sequence (with three associated transitions for each light and labeled surrogate) for the 11 noncatalytic subunits (α1–7; β3, β4, β6, β7) (supplemental Data S3) was applied. With this method, absolute quantification of total 20S proteasome was achieved in protein lysates of eight different human cell lines of diverse origins (HEK 293T, HCT116, RKO, U937, HeLa S3, NB4, KG1a, and MRC5). 20S proteasome concentration determined by the SRM method was compared with those obtained with the reference ELISA method (Fig. 2*A*). A high correlation was found between the two methods (coefficient of determination = 0.98; slope = 1.01). Altogether, these data demonstrate that the newly-developed method is suitable for absolute quantification of 20S proteasome in human cell lysates.

Next, we implemented the method to detect the six standard and immune-specific catalytic subunits of the 20S proteasome (β1, β2, β5, β1i, β2i, β5i), as the absolute quantification of these subunits is required to determine the stoichiometry of the different 20S proteasome subtypes present in biological samples (Fig. 1). Two to three proteotypic peptide sequences (and three or four associated transitions for each light and labeled surrogate) were carefully chosen, paying attention to favoring wide distribution over the protein sequence. Transitions and detection were then optimized for the six catalytic subunits. In total, the final method to quantify all α and β subunits comprised 206 independent MS transitions (103 “light” transitions and 103 “heavy” transitions corresponding to heavy surrogate peptides) associated with optimized dwell times and voltages for detection by the mass spectrometer (supplemental Data S3). The method to quantify of the absolute levels of all six 20S proteasome catalytic subunits was validated by taking advantage of the known stoichiometry of incorporation of catalytic subunits into the 20S proteasome (supplementary Information I-2). Based on the excellent accuracy (97 ± 2%) and variability (CVs below 15%) of the method to measure the absolute levels of catalytic subunits (supplementary Information I-2 and supplemental Fig. S7*B*–S7*D*), these data were further used to determine changes in the stoichiometries of the four 20S proteasome subtypes observed in a model of IFNγ-treated HeLa cells (sP20S, β5i P20S, β1iβ5i P20S, and iP20S - no β5t P20S nor α4s P20S detected) (as detailed under “Experimental procedures”) (Fig. 2*B*). As expected, iP20S was highly and significantly increased (by a factor of 12), becoming the major proteasome subtype after 96 h stimulation with IFNγ, whereas sP20S levels dropped by nearly 3-fold (Fig. 2*B*). Strikingly, the two intermediate 20S immunoproteasome subtypes, β5i P20S and β1iβ5i P20S, which each represented around 10% of the total 20S present, did not vary significantly after the cytokine stimulation. This interesting result shows that the main impact of IFNγ stimulation on the composition of proteasome complexes is the progressive replacement of the sP20S by the iP20S subtype.

##### Determining the Stoichiometry of 20S Subtypes in Human Tissues of Broad Origins

**Fig. 3.**
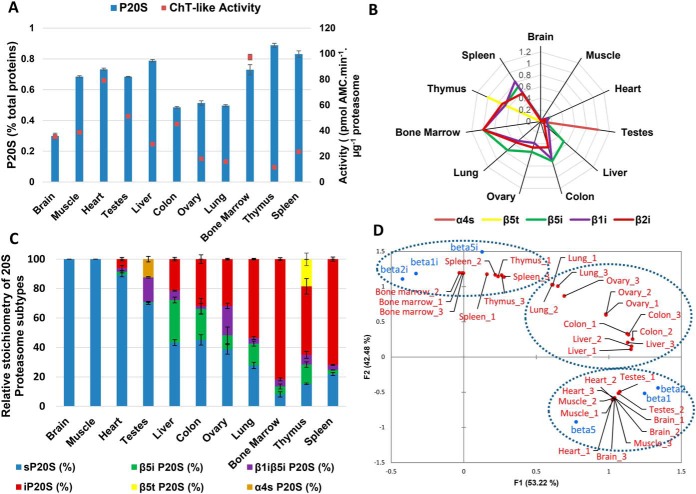
**The method is able to precisely and accurately monitor 20S proteasome absolute quantities and subtypes stoichiometries in a panel of human tissues.** *A*, Proteasome 20S absolute quantity (% w/w total proteins) measured by the LC-SRM method and 20S Proteasome chymotrypsin-like activity measured by degradation of LLVY-AMC peptide, in a panel of 11 human tissues. *B*, Label-free relative quantification of tissue-specific 20S proteasome subunits by label-free LC MS/MS reveals the tissue-specificity of α4s and β5t subunits but not the one of immunocatalytic subunits. Relative abundances of each subunits across the tissues are represented. The highest abundance is arbitrarily set to 1. *C*, Stoichiometries of the six proteasome 20S subtypes in different Human tissue determined by the LC-SRM method. Details on stoichiometry determination of 20S proteasome subtypes are provided under Experimental procedures. *D*, Biplot Principle Component Analysis of 11 human tissue samples (3 replicates per tissue) based on 20S proteasome catalytic subunits composition. The dashed circles represent the main clusters observed depending on content on standard (β1, β2, β5) or immuno (β1i, β2i, β5i) subunits.

Next, we applied the method to determine the absolute quantities and precise stoichiometries of 20S proteasome subtypes in 11 protein lysates extracted from human tissues of broad origins (Fig. 3). In most tissues, the proteasome represented 0.2 to 1% of the total protein present (Fig. 3*A*). A shotgun label-free LC-MS/MS proteomics analysis exclusively identified and quantified α4s and β5t in testes and thymus samples, respectively, confirming their tissue-specificity (4), whereas the immunocatalytic subunits (β5i, β1i, and β2i) were much more widely distributed across tissues (Fig. 3*B*). The stoichiometries of the six major 20S proteasome subtypes (Fig. 1*A*) were determined based on the absolute quantities of α and β subunits measured using the optimized method (as explained under Experimental Procedures). The absolute level of the catalytic β5t subunit provided the abundance of the thymoproteasome (Fig. 1*A*). Because this protein is not incorporated into labeled sP20S and iP20S internal standards, the absolute quantity of β5t was deduced from absolute amounts of total P20S, β5 and β5i (Fig. 3*C*). Similarly, the α4s subunit (PSMA7L protein) which is representative of the spermatoproteasome could not be directly quantified. Although α4 and α4s are very similar in sequence, three peptide sequences that are specific to the PSMA7 protein (α4) were detectable and were used to distinguish between the two isoforms (supplemental Fig. S8*A*). The absolute level of α4s was deduced from standard P20S and α4 contents (Fig. 1*B*, Experimental Procedures). As in the shotgun assay, proteotypic peptides corresponding to the thymoproteasome and spermatoproteasome were only observed in thymus and testes tissues, respectively, (Fig. 3*B*) and these two 20S proteasome subtypes represented only 20 and 12% of the total proteasome pool in these organs, respectively (Fig. 3*C*). A principal component analysis (PCA) biplot based on catalytic subunit composition (Fig. 3*D*) grouped brain, muscle, heart, and testes tissues as they almost exclusively contain standard proteasome β subunits (β1, β2, and β5). Bone marrow, spleen, and thymus were observed on the opposite side of the graph because they contain high levels of β1i, β2i, and β5i immunosubunits. Interestingly, intermediate 20S subtypes (β5i P20S and β1iβ5i P20S) were only detected in low abundance (less than 10% of the total 20S proteasome pool) in bone marrow and spleen, suggesting that the immunoproteasome is the most important proteasome subtype for antigen processing among immunosubunit-containing subtypes. Liver, colon, ovary and lung tissues were placed at an intermediary position on the PCA graph (Fig. 3*D*), with 50 to 70% of immunosubunit-containing 20S proteasome subtypes (Fig. 3*C*).

Interestingly, the absolute amount of the α4 subunit, measured using peptides common to PSMA7 and PSMA7L proteins (supplemental Fig. S8*A*–S8*B*), was stoichiometric and highly correlated with the total 20S proteasome amount (measured using the α and β noncatalytic subunits, apart from α4) both in the panel of eight cell lines (R^2^ = 0.97; slope = 0.99) and in tissues of diverse origins (R^2^ = 1.00; slope = 1.01) (supplemental Fig. S8*C–*S8*D*). This result once again confirms the accuracy and precision of the method developed while also emphasizing the need for absolute quantification to detect perturbations in proteasome subunit expression or incorporation of subunits into mature proteasomes. It also indicates that this broad panel of normal and tumor human cells do not contain the noncanonical alternative α4-α4 20S proteasome complex in which a second copy of α4 occupies the position normally held by α3 (38, 39).

The abundance of some proteasome subunits measured using our optimized MRM assay was compared with mRNA expression data retrieved from the human protein atlas database (40) in corresponding tissues (supplemental Fig. S9). Although positive correlations were observed between some proteins and mRNA molecules abundances, these results suggest that measuring mRNA levels are not suitable when seeking to predict protein expression levels for 20S proteasome subunits in human tissues.

##### Proteasome Status Is Affected by Priming ADSCs with IFNγ During Their Ex Vivo Expansion

**Fig. 4.**
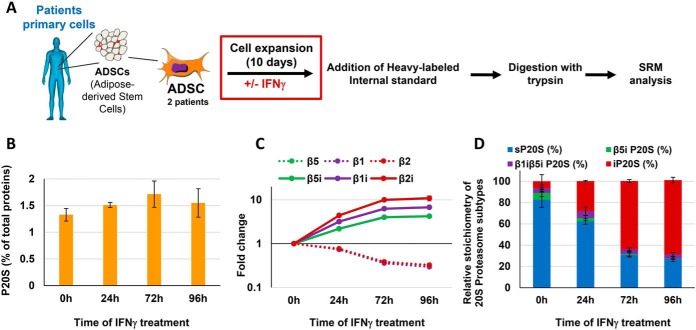
**Proteasome status is affected by priming ADSCs with IFNγ during their *ex vivo* expansion.** *A*, Workflow for the determination of the effect of priming of ADSCs with interferon-γ on their 20S proteasome status, using the LC-SRM method. Primary cells obtained from two patients were used for the study. *B*, Proteasome 20S absolute quantity (% w/w total proteins) measured by the developed LC-SRM method in ADSCs obtained from patients (*n* = 2 biological replicates - 3 technical replicates). *C*, Fold change in the six catalytic subunits of the 20S proteasome during IFNγ stimulation. ADSCs were cultivated in the presence of the cytokine (100 ng/ml). Absolute quantities of the catalytic subunits were determined by the developed LC-SRM approach and fold changes are calculated relative to time 0h (*n* = 2 biological replicates − 3 technical replicates). *D*, Dynamics in the stoichiometries of 20S proteasome subtypes during IFNγ stimulation of primary ADSCs derived from patients (*n* = 2 biological replicates − 3 technical replicates). Calculations are detailed under Experimental procedures.

Thanks to their immunosuppressive properties, MSCs are considered a promising tool for cell therapy. Much effort has been directed toward enhancing MSCs activity by treatment with IFNγ. However, any *ex vivo* modifications may also fundamentally alter the cells, and understanding determinants that affect their immunomodulatory activity is essential if we are to develop effective MSC strategies. MSCs obtained from patients must be expanded *ex vivo* if they are to be used in the clinic, and this process must be carefully controlled (41). Recently, we reported a decrease in the immunosuppressive properties of ADSCs cultured up to the senescent stage and demonstrated that this effect was the result of proteasome-mediated indoleamine 2,3 dioxygenase (IDO) degradation (42). Priming of MSCs with IFNγ has been shown to extensively potentiate their therapeutic activity (43, 44) but, to our knowledge, and although proteasome functions cover a broad spectrum of biological functions, the proteasome status has never been assessed in this context. When used to measure the consequences of IFNγ-stimulation of *ex vivo*-expanded patient-derived ADSCs on proteasome status (Fig. 4*A*), our method revealed no major changes in the total 20S proteasome quantity (Fig. 4*B*). However, a massive replacement of standard catalytic subunits by their immunological counterparts was clearly observed (Fig. 4*C*), resulting in a strong shift from the standard to the immunoproteasome subtype (Fig. 4*D*). Indeed, although the standard subtype represented 75% of the 20S proteasome pool when ADSCs were expanded in an IFNγ-free medium, this proportion was decreased to 25% after 4 days' culture in the presence of the cytokine (Fig. 4*D*). The massive induction of the immunoproteasome by cytokine stimulation is likely to have a considerable impact on the repertoire of degraded proteins and antigenic peptides loaded onto major histocompatibility complex class I (MHCI) molecules.

##### Expansion of ADSCs Under Different O_2_ Concentrations Affects 20S Proteasome Status and Their Capacity to Differentiate

Conventionally, MSC culture for clinical applications is performed under normoxic conditions (21% oxygen tension), even though oxygen levels within tissues are typically much lower (hypoxic) than these standard culture conditions. Therefore, oxygen tension represents an important environmental factor that may affect how MSCs perform *in vivo*. However, the impact of hypoxic conditions on distinct mesenchymal stem cell characteristics, such as the proteasomal status, remains unclear.

**Fig. 5.**
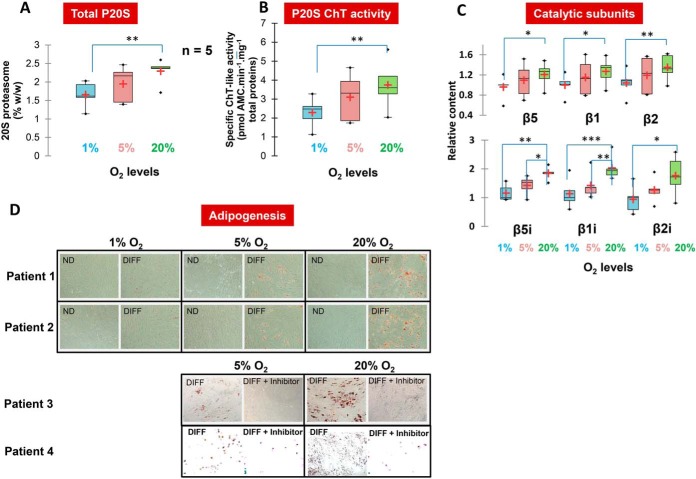
**Expansion of ADSCs in different O_2_ levels affect 20S proteasome status and ADSCs' capacity to differentiate.** *A*, Proteasome 20S absolute quantity (% w/w total proteins) measured by the LC-SRM method in five patients derived primary ADSCs cultivated at three different levels of O_2_ (1%, 5%, and 20%). *B*, Proteasome 20S chymotrypsin-like activity measured by the *in vitro* degradation of the LLVY-AMC peptide by proteasome in ADSCs lysates after cultivation in three different levels of O_2_ (1%, 5%, and 20%) (*n* = 5 patients − 3 technical replicates). *C*, Relative content of standard and immuno catalytic 20S proteasome subunits measured by the method in five patients derived primary ADSCs cultivated at three different levels of O_2_ (1%, 5%, and 20%). *D*, Differentiation potential of human ADSCs from different patients under different levels of O_2_ (1%, 5%, and 20%). The immunoproteasome inhibitor ONX-0914 alters the differentiation capacity of ADSCs at 5 and 20% O_2_ concentrations. Adipogenic differentiation is indicated by Oil red O staining.

We applied our method to analyze the 20S proteasome status after 10 days of ADSCs expansion under three different oxygenation concentrations: 1%, 5%, and 20%. Interestingly, proteasome abundance, proteolytic activity and composition were all affected by dioxygen levels (Fig. 5*A*–5*C*). Indeed, when comparing the effect of ADSC expansion in hypoxic or normoxic conditions (1% *versus* 20% O_2_ levels), we detected a significant increase in total 20S proteasome abundance alongside a nearly 2-fold significant upregulation of the three immunoproteasome catalytic subunits (Fig. 5*A* and 5*C*). Both changes probably account for the higher 20S proteasome chymotrypsin-*like* activity measured following culture in normoxic conditions (Fig. 5*B*). Indeed, structural differences in active sites account for the higher chymotrypsin-*like* activity of the β5i subunit of the immunoproteasome compared with its β5 standard counterpart (5, 45). Moreover, the relative abundance of 20S proteasome-associated PA28αβ activator is decreased in hypoxia compared with normoxia (*p* = 0.02) whereas the O_2_ level had no significant impact on the level of association of the other 20S CP regulators (supplemental Fig. 10*B*). This last observation further supports the increase measured in immunoproteasome subtype in normoxic compared with hypoxic conditions as PA28αβ is known to preferentially associate with the 20S immunoproteasome variant *in cellulo* (34).

Overall, in the absence of IFNγ in the culture medium, the major proteasome subtype observed in ADSCs was the standard proteasome, which represented 60 to 80% of the total proteasome pool, depending on patient and O_2_ level (supplemental Fig. S9*A*). Given the massive induction of immunoproteasome upon exposure to the pro-inflammatory cytokine (Fig. 4*C*) and the absence of immune cells in the ADSC culture medium, the inter-individual differences observed in immunoproteasome content might result from variability in the inflammatory context before surgery. Immunoproteasome subunits have been shown to be required for adipocyte differentiation (29). Accordingly, our data indicate that high levels of immunoproteasome correlate with high adipogenic potential at 20% O_2_, and the reverse under hypoxic conditions (Fig. 5*D*). Strikingly, pharmacological inhibition of the immunoproteasome using the ONX-0914 inhibitor (46) leads to a marked decrease in the capacity of ADSCs to differentiate into adipocytes, which confirms previous observations in the PSMB8 KO mice (29) using an orthogonal method.

To get a broader picture of the effect of O_2_ levels on the proteome of ADSCs, a whole proteome analysis was performed on ADSCs grown under normoxic or in hypoxic conditions (1% of O_2_). Importantly, a new cohort of three patients was used to obtain this new biological material. Out of 3624 proteins identified and quantified, 67 proteins were found at increased levels in hypoxic conditions and 129 were over-represented in normoxic conditions (supplemental Fig. S11 and revised supplemental Data S7). All three immunocatalytic subunits were increased under normoxic conditions (fold increase of 1.6 to 1.9) although statistical significance was only reached for β1i and β2i (*p* value = 0.05). As discussed above (supplemental Fig. S7), this apparent discrepancy can easily be explained by the lower precision of label-free quantification results compared with SRM quantification data.

Functional analysis based on GO Biological processes revealed the most significant pathway related to the proteins for which abundance was modified both under hypoxic and normoxic conditions to be the “oxidation-reduction” pathway (FDR = 1.2E-06 and 5.9E-03 for proteins upregulated in hypoxic and in normoxic conditions, respectively). This result was to be expected following a change in O_2_ levels from 20% to 1%. Other enriched GO Biological pathways included “response to oxidative stress” (FDR = 4.2E-02) in the proteins that were more abundant in normoxic conditions. This pathway is particularly interesting as the immunoproteasome has been associated with responses to oxidative stress (47–49). ADSCs were also shown to negatively regulate oxidative stress *in vivo* (50) and notably through Nrf2 which is one of the main transcription factors involved in controlling oxidative stress. In our whole-proteome analysis, we observed that NQO1 (2-fold increase, *p* value = 0.02), one of the best-known targets of Nrf2 (51), was increased under normoxic compared with hypoxic conditions. Reactive oxygen species seem to play an important role in adipocyte differentiation (52–54). A function of the immunoproteasome related to ADSC differentiation could be the clearance of proteins having undergone oxidative damage, when this type of damage accumulates it could be toxic for the cell. Significantly, 25 of the proteins for which overall abundance differences were detected are functionally related to the “cell differentiation” GO Biological Process (FDR = 1.4E-02 for proteins over-represented in cells grown under hypoxic conditions). Among these, 16 were increased in the hypoxic cells whereas 9 were decreased. These proteins are listed in supplemental Data S7 (4th sheet). Given the importance of the immunoproteasome in regulating ADSC differentiation when stimulated with dexamethasone, and because our results clearly demonstrate that the immunoproteasome is increased under normoxic compared with hypoxic conditions, it is tempting to speculate that some of these deregulated proteins might be direct or indirect targets of the immunoproteasome. In particular, Transforming growth factor-beta-induced protein ig-h3 (TGFBI/BGH3) and Plasminogen activator inhibitor 1 (PAI1) were both increased in our hypoxic cells (4.1- and 2.9-fold increase, respectively, with *p* values of 8.5E-4 and 0.03, respectively), and these proteins are known to be target genes of TGFβ1, a pathway required for hypoxia-mediated inhibition of adipocyte differentiation (55). Of course, further experiments will be necessary to confirm that these proteins are downstream players of the immunoproteasome affecting the adipogenic potential of ADSCs.

In conclusion, after as few as 10 days of ADSCs expansion under different dioxygen levels, the method developed could detect mild but still significant changes in 20S proteasome status, and variations in the level of 20S immunoproteasome. This change in proteasome composition might be causal for the observed O_2_-induced variations in differentiation potential of ADSCs even though further investigation will be required to determine the precise mechanisms.

## DISCUSSION

This study aimed to develop an assay to determine subunit absolute quantity and stoichiometry within a highly heterogeneous macromolecular protein complex, the 20S proteasome. To achieve this goal, a robust, accurate and sensitive absolute quantification method with multiplexing ability had to be developed to allow the absolute quantification of the 19 different subunits of the 20S proteasome in a single assay.

Absolute quantitation based on IDMS using the SRM scanning mode suits the requirements for multiplex detection and is also recognized for its specificity, sensitivity, and accuracy (16, 17). Because many proteasome subunits had to be analyzed, we first used the most straightforward IDMS proteomics strategy, the AQUA (18) method, which involves spiking various isotope-labeled synthetic peptides into samples as internal standards. Although mainly used for the absolute quantification of monomeric proteins, the AQUA method has also been successfully applied to assess the sub-stoichiometric incorporation of the Rpn13 ubiquitin receptor within the 26S proteasome (56). Our AQUA peptide sequences were carefully chosen to meet the requirements for IDMS analysis of proteins (16) and, when tested in simple or more complex biological matrices, they provided good linearity in signal response, low variability, and adequate sensitivity. These analytical performances explain why the AQUA technology was so rapidly adopted for the LC-SRM validation of protein biomarkers in various biological matrices (16, 17). However, we noted a defect in trueness that was easily detected in our system because noncatalytic proteasome subunits are expected to be present at equimolar stoichiometry. Responses were observed to be highly dependent on peptide sequences, probably because of incomplete enzymatic digestion of the protein of origin; other reasons for the discrepancy observed could be incomplete peptide solubilization, peptide instability, or artifactual chemical modifications. Thus, isotope-labeled peptides could not be used to accurately assess proteasome subtype stoichiometry unless laborious optimizations were undertaken (19). Several IDMS methods based on the use of isotope-labeled protein standards like PSAQ (21), absolute SILAC (22), PrEST (23), or TAQSI (25) were developed to overcome the issues of the AQUA methodology. To the best of our knowledge, none of these has yet been applied to determine protein complex stoichiometry. To achieve this goal on highly heterogeneous 20S proteasome complex, we optimized a workflow relying on SILAC-based absolute quantification. A reference mixture containing equimolar concentrations of both isotope-labeled and purified 20S standard- and immuno-proteasome subtypes was produced, qualitatively and quantitatively assessed, and spiked into protein lysates extracted from cell cultures or tissues. After protein digestion, the SRM method was optimized for carefully chosen proteotypic peptides. In this approach, use of a whole isotope-labeled protein as internal standard represents a clear advantage over the PrEST approach (23) because all proteotypic peptides can theoretically be used for quantification. This method was validated by comparison with the reference ELISA method for total 20S proteasome absolute quantification in a broad range of biological samples.

Although 20S and 26S proteasome complexes can be routinely quantified using different ELISA assays (6, 10–14, 33), this technique lacks the multiplexing capacity to assess the complete diversity of proteasome subtypes because up to 19 different protein chains can be incorporated into the 20S proteasome macromolecular assembly (Fig. 1*A*). Moreover, to our knowledge, the precise stoichiometries of tissue-specific β5t- and α4s-containing 20S proteasome subtypes have never yet been determined. Using our optimized assay, for the first time, we accurately quantified the stoichiometry of the tissue-specific thymoproteasome and spermatoproteasome; their low levels in thymus and testes tissues (20 and 12% of the total 20S proteasome pool, respectively) support findings in previous reports showing that β5t and α4s are detected in a subpopulation of cells in these two organs (4, 58).

Then, we demonstrated that our method could also precisely monitor changes in 20S proteasome β catalytic subunit composition following IFNγ-activation of HeLa cells. We took advantage of the known stoichiometry of β catalytic subunits in the 20S proteasome to assess the trueness of the method, which exceeded 96%. In comparison, the TOP3 label-free quantification approach barely achieves 78% accuracy after discarding outliers arising from unrepresentative peptides. Converting peptide abundance into protein concentration requires careful selection of peptide sequences and optimization of SRM transitions (19) or, alternatively, the removal of incoherent peptides from global MS1-based quantification datasets, for instance using covariation of peptide abundances (59). Thus, even if label-free MS1-based quantification methods can be used to obtain a rough estimate of protein complex stoichiometry, as previously reported (60–62), these approaches are most appropriate for the high-throughput determination of changes in the relative abundances of protein complex subunits (34, 35) or estimation of protein copy-number without requiring spike-in standards (63).

MSCs obtained from bone marrow (BM-MSCs) or AT (ADSCs) are promising tools for cell therapy in regenerative medicine, to treat severe inflammatory and autoimmune diseases, or to prevent transplant rejection (64). Cells must be expanded *ex vivo* to obtain enough numbers for use in therapy. Thus, culture conditions must be carefully controlled both for safety issues and to optimize therapeutic effectiveness. Using our method, we showed that pre-stimulation of ADSCs with IFNγ and increasing O_2_ levels both affect the status of the 20S proteasome during *ex vivo* expansion of primary ADSCs. In both cases, changes in proteasome composition were observed, an increase in immunoproteasome stoichiometry. These results emphasize the high plasticity of the 20S proteasome when exposed to external stimuli, but also the multiple biological roles played by the immunoproteasome subtype. IFNγ is known to induce the immunoproteasome through the formation of new proteasome particles incorporating immunocatalytic subunits in place of the standard ones. Pre-stimulation of both BM-MSCs and ADSCs with pro-inflammatory cytokines, IFNγ, has been shown to increase their immunosuppressive properties (43, 64, 65), by increasing release of several soluble immunosuppressive factors, in particular kynurenine, a product of indoleamine 2,3-dioxygenase (IDO) activity (65). Moreover, both BM-MSCs and ADSCs are antigen presenting cells (64) and, when exposed to IFNγ, they upregulate expression of HLA class I molecules on their surface. This response protects them from NK-mediated lysis (43, 66), and promotes their immunomodulatory effects. Strikingly, both mechanisms linked to the immunosuppressive properties of MSCs are affected by proteasome status. Indeed, a shift from standard to immunoproteasome considerably increases the production of MHC class I-binding peptides *in vivo* (67) and IDO is known to be a proteasome substrate (68). Proteasome-mediated degradation of IDO was recently reported to explain the reduction in immunosuppressive potential observed in clinical-grade expanded MSCs which had reached replicative senescence (42). Mechanistically, IFNγ is known to increase IDO protein via transcriptional activation (69) but it is also responsible for a strong induction of the immunoproteasome-PA28αβ complex, a proteolytic system functioning in the absence of ubiquitination (67). As IDO is degraded through the ubiquitination-dependent SOC3-proteasome pathway (68), the increase in ubiquitin-independent degradation triggered by IFNγ might constitute a mechanism further promoting IDO stabilization in this physiological context.

In addition to its major role in antigen processing, the immunoproteasome seems to degrade oxidized proteins more efficiently than the standard 20S CP (70, 71). *De novo* synthesis of 20S proteasome and of immunoproteasome is crucially important for maintaining efficient proteostasis in oxidative stress conditions (49). The evidence provided here of low total proteasome content and an absence of immunoproteasome in brain probably accounts for the previously reported higher sensitivity of brain tissues to oxidative stress (72). Activation of the immunoproteasome and autophagy occur during the early stages of ESC differentiation, to allow degradation of damaged proteins and avoid their transmission to differentiated cells (27). Moreover, recent findings indicate that the immunoproteasome is required for differentiation of adipocytes (29) and skeletal muscle (28). In line with this requirement, our results demonstrate that ADSCs grown under hypoxic conditions, had a lower immunoproteasome content and exhibited a lower differentiation potential than their counterparts grown under 20% O_2_. In addition, our results suggest that immunoproteasome activity is causal for the change in adipogenic potential in normoxic conditions. Thus, the significant increase observed in all three immunocatalytic subunits and in overall proteasome activity in normoxia-cultured primary ADSCs compared with the cells cultured in hypoxic environments could reflect modulation of their therapeutic capacities. Importantly, ADSCs are adult pluripotent stromal stem cells isolated from white AT where O_2_ levels are below 5% but they are routinely expanded *ex vivo* under 20% O_2_. Manufacturing practices for ASC expansion must therefore be carefully optimized and controlled to maintain their therapeutic capacity. The results from the approach developed here applied to primary ADSCs grown in conditions close to real clinical production clearly demonstrate that IFNγ and dioxygen levels could be key parameters in this process.

To conclude, the method presented here allows robust and rapid determination of the complete status, *i.e.* absolute quantity and stoichiometry, of a highly heterogeneous macromolecular protein complex, the 20S proteasome, in various human tissues and cells. When applied to primary ADSCs expanded in different culture conditions, our results highlighted a high plasticity in proteasome composition and abundance which might be related to modulation of the ADSCs' immunosuppressive and differentiation properties. The method developed thus constitutes a sound approach to complement immunophenotyping or other methods to monitor protein markers (73) for the optimization and control of manufacturing processes for ADSCs expansion. Knowledge of proteasome composition is also of major interest for therapeutic purposes. Indeed, upregulation or dysregulation of immunoproteasome catalytic subunits have been observed in several human diseases and disorders, such as inflammatory and autoimmune diseases, cancer, diseases of the central nervous system, and aging (74). For instance, mutations reported in immunocatalytic subunits and the resulting defects in 20S immunoproteasome assembly and activity observed in PRAAS (Proteasome-Associated Autoinflammatory Syndrome) patients could be monitored using our method (reviewed in (75)). Our assay could also be of great interest when seeking to assess patients before instigating immuno-therapy as the efficacy of tumor antigen processing and presentation is closely linked to levels of 20S immune-type proteasomes in antigen presenting cells (75). Detection of increased concentrations of 20S proteasomes or changes in subtype profiles could also be monitored in extracellular body fluids to diagnose and/or as a prognostic marker of various diseases (76). In this context, a more specific targeting of the different heterogeneous forms of the proteasome will lead, in the long run, to more specific treatments generating fewer side effects and less chemoresistance than caused by broad-spectrum proteasome inhibitors (77). However, to design such targeted therapies, tools to precisely determine patients' proteasome status, like the one presented here, will need to be developed.

More generally, the developed strategy could be extended to assess the absolute level, dynamic, and heterogeneous nature of many other biologically-relevant macromolecular systems such as the human spliceosomal hprp19/CDC5L complex (19, 61), the nuclear pore complex (78), core ribosomal proteins (62), or even host-pathogens interactions (79). These adaptations would be more accurate than the peptide-based mass spectrometry methods currently used (61). The sole requirement for this adaptation is that it must be possible to ectopically produce and purify each individual subunit in carefully-controlled absolute quantities with heavy-isotope incorporation.

## DATA AVAILABILITY

The data sets corresponding to the mass spectrometry analyses presented in this study have been deposited in the following repositories: PRIDE, (https://www.ebi.ac.uk/pride/archive/, Project accession: PXD011894) for label-free MS analyses, and PeptideAtlas (http://www.peptideatlas.org/, Dataset identifier: PASS01219) for targeted MS analyses. Skyline files of all targeted experiments are available on Panorama Public (https://panoramaweb.org/project/Panorama%20Public/2018/IPBS-CNRS%20-%20SRM_Proteasome_2018/begin.view?). The detailed descriptions of all analyses (raw and processed file names, sample name, biological replicate number, MS technical replicate number, corresponding figure) are summarized in Supplementary Data 8.

## Supplementary Material

supplemental Data S3

Supplementary Information

Supplemnatry Figures

Peptides sequences, SRM transitions, and voltages applied for the LC-SRM analysis of 20S Proteasome using AQUA peptides

Peptides sequences, SRM transitions, and voltages applied for the LC-SRM analysis of 20S Proteasome using isotope-labeled whole proteasome complex

Peptides sequences, SRM transitions, and voltages applied for the LC-SRM analysis of 20S Proteasome using isotope-labeled whole proteasome complex

Peptides sequences, SRM transitions, and voltages applied for Quality Controls (QC)

Experimental LOD and LLOQ

Protein and Peptide identification data corresponding to Figure 3B

Protein and Peptide identification data corresponding to supplementary Figure 11

Detailed description of mas spectrometry data sets deposited in repositories

## Footnotes

^1^ The abbreviations used are:

sP20S: standard 20S proteasome
iP20S: immunoproteasome
β5t P20S: thymoproteasome
α4s P20S: spermatoproteasome
ADSCs: Adipose-derived Mesenchymal Stromal/Stem Cells
AT: Adipose Tissue
CID: Collision Induced Dissociation
FBS: Fetal Bovine Serum
IDMS: Isotope Dilution Mass Spectrometry
IFNγ: interferon γ
vMSCs: Mesenchymal Stem Cells
SILAC: Stable Isotope Labeling by Amino acids in Cell culture
SRM/MRM: Single Reaction Monitoring/Multiple Reaction Monitoring
SVF: Stromal vascular fraction.
