# Supplementary material for: Mass Spectrometry-based Absolute Quantification of 20S Proteasome Status for Controlled Ex-vivo Expansion of Human Adipose-derived Mesenchymal Stromal/Stem Cells
Source: Mol Cell Proteomics. 2019 Jan 30;18(4):744–59. doi: 10.1074/mcp.RA118.000958 (PMC6442357; doi:10.1074/mcp.RA118.000958)
Supplement: supplemental Data S3 [file RA118.000958_index.html]

Supplement to Mass spectrometry-based absolute quantification of 20S proteasome status for controlled ex-vivo expansion of Human Adipose-derived Mesenchymal Stromal/Stem Cells | Molecular & Cellular Proteomics

## Supplemental Data

- Supplementary Information - Contains : I- Supplementary Results, II- Supplementary Figures Legends, III- Supplementary Data List and Legends and IV- References
- Supplemnatry Figures - Supplementary Figures 1 to 11. Supplementary Figures legends are provided in the Supplementary Information file\_Part II
- Peptides sequences, SRM transitions, and voltages applied for the LC-SRM analysis of 20S Proteasome using AQUA peptides - Supplementary Data corresponding to corresponding to Sup Fig. 1 & 2
- Peptides sequences, SRM transitions, and voltages applied for the LC-SRM analysis of 20S Proteasome using isotope-labeled whole proteasome complex - Supplementary Data corresponding to corresponding to Sup Fig. 4 & 5
- Peptides sequences, SRM transitions, and voltages applied for the LC-SRM analysis of 20S Proteasome using isotope-labeled whole proteasome complex - Supplementary Data corresponding to corresponding to Figures 2-5 and Sup Fig. 8 & 9
- Peptides sequences, SRM transitions, and voltages applied for Quality Controls (QC) - QC : injection of 20 fmol of tryptic digest of betagalactosidase
- Experimental LOD and LLOQ - Experimental LOD and LLOQ obtained by injecting heavy-isotope labelled sP20S and iP20S spiked at increasing concentrations in a HeLa protein lysate. The QuaSAR tool, available through the Skyline interface, was used to generate LOD and LLOQ values.
- Protein and Peptide identification data corresponding to Figure 3B - Label-free relative quantification of tissue-specific 20S proteasome subunits in 11 human tissues by label-free LC MS/MS. Data corresponding to Figure 3B.
- Protein and Peptide identification data corresponding to supplementary Figure 11 - Label-free relative quantification of ADSCs proteins grown Under hypoxic and normoxic conditions
- Detailed description of mass spectrometry data sets deposited in repositories - raw and processed file names, sample type, biological replicate number, MS technical replicate number, analytical conditions
