# Supplementary material for: Mass Spectrometry-based Absolute Quantification of 20S Proteasome Status for Controlled Ex-vivo Expansion of Human Adipose-derived Mesenchymal Stromal/Stem Cells
Source: Mol Cell Proteomics. 2019 Jan 30;18(4):744–59. doi: 10.1074/mcp.RA118.000958 (PMC6442357; doi:10.1074/mcp.RA118.000958)
Supplement: Supplementary Information [file 139223_1_supp_270974_plj9nm.pdf]

Supplementary Information

Contains : I- Supplementary Results, II- Supplementary Figures Legends, III- Supplementary Data List and Legends and IV- References

I. Supplementary Results

1- Assay design and choice of the IDMS approach for determination of total 20S proteasome absolute quantity

The first step of the assay development was to design and validate a robust quantification for total 20S proteasome content using SRM analysis. In a first attempt to assess the absolute 20S proteasome quantity in biological samples using SRM analysis, an isotope dilution method relying on heavy-labeled internal standard peptides (AQUA peptides) was developed (Figure 1B). On the basis of « golden rules » for the selection of proteotypic peptide sequences (1), and preliminary experiments to optimize transitions and voltages on the Q-Trap instrument (DP, EP, CE, CXP), a method containing 60 transitions for endogeneous (light) and heavy-labeled (AQUA) peptide sequences and corresponding to four 20S proteasome non-catalytic subunits (α3, α4, β6, and β7) and six 20S proteasome catalytic subunits (β1, β2, β5, β1i, β2i, and β5i) was set (Supplementary Data 1; supplementary Figure 1A). The four peptides corresponding to the non-catalytic subunits are expected to give similar quantitative responses as these subunits are stoichiometrically incorporated into all 20S proteasome subtypes. The absolute level of total 20S proteasome pool can also be obtained from these measured quantities. Moreover, these peptides were carefully chosen not to be specific of some subunit isoforms. For example, the ALLEVVQSGGK peptide corresponding to the α4 subunit is shared between the ubiquitously α4 protein (PSMA7 protein) and its testes-specific isoform α4s (PSMA7L protein), and therefore represents the summed quantities of these two α4 isoforms. This method showed an excellent linearity over a range of three orders of magnitude, from 0.5 to 500 fmol on column (coefficients of determination above 0.99) and a very good reproducibility (mean CV < 10%), even at the lowest quantity range (0.5-5 fmol injected on column), as assessed by injection of commercial proteasomes purified from human

erythrocytes (Supplementary Figure 1B-C). Then, to evaluate the method for proteasome absolute quantification in real cellular extracts, the same commercial 20S proteasome preparation was spiked at five different concentrations in a U937 cells protein lysate previously depleted from endogeneous proteasome (using proteasome immuno-purification (2)). Proteasome concentration was then measured by the ELISA reference method or by using the SRM approach. The SRM responses of the four non-catalytic subunits peptides show very good linearities over the concentration range studied (10 to 100 femtomoles injected on column, corresponding to proteasome useful working concentrations in the protein lysate of 2 to 20 mg/mL) (Supplementary Figure 2A) and reproducibilities (CV max = 9.4 %) (Supplementary Figure 2C). Despite these high analytical performances, discrepancies were observed across peptides concerning the absolute quantity measured for total 20S proteasome, highlighting a lack of accuracy of the method (mean accuracy of 41.5 %, Supplementary Figure 2C). Three peptide sequences (LLDEVFFSEK, ALLEVVQSGGK, GAVYSFDPVGSYQR) under-estimate the absolute quantity of 20S proteasome given by the reference ELISA method (Supplementary Figure 2A). This problem might arise from an incomplete tryptic digestion of the endogeneous protein, as discussed in many other studies (3–5). On the contrary, the AIHSWLTR peptide sequence overestimates the absolute proteasome concentration (Supplementary Figure 2A), possibly because of incomplete solubilisation or partial oxidation (W site) of the AQUA peptide. Overall, these results illustrate the difficulty of using peptide-based isotopic dilution to absolutely quantify proteins. The excellent reproducibility observed allow relative quantification, which is the final goal of main studies relying on SRM. However, as far as absolute quantity or stoichiometry assessment is needed, accurate quantification is a critical requirement.

To overcome this analytical issue, we moved to a more robust quantification strategy based on the isotope labeling of the full-length protein. Quantification of the different 20S proteasome subtypes requires the production and purification of several isotope-labeled protein standards of non-catalytic and catalytic subunits. Though PSAQ standards typical production systems like cell-free systems or bacteria could have been applied, we developed a more direct approach where pure and isotope-

labeled 20S standard- and immuno- proteasomes were produced and purified from human 293-EBNA cells which express sP20S, or 293-EBNA cells engineered to express iP20S (Supplementary Figure 3). These cell lines were previously obtained by transfecting 293-EBNA cells with cDNAs encoding the three immuno catalytic subunits β5i, β1i and β2i, as already published (6). The non-transfected cells express mainly the three catalytic subunits β5, β1 and β2 of sP20S, whereas the transfected cells produces only the counterpart catalytic subunits of iP20S, β5i, β1i and β2i, respectively, as shown using immuno-detection of these subunits (Supplementary Figure 3). Cell cultures were performed in media containing the heavy forms of arginine, R($^{13}C_6$), and lysine, K($^{13}C_6$). Ten cellular doubling were performed in this medium to reach a 95% rate of heavy amino acids incorporation into proteins (assessed by MS). Standard 20S proteasome and iP20S were then purified as described earlier (2). Absolute quantities and purities of both purified proteasome subtypes were then assessed as follow. The approach consisted in the spike-in of a known quantity of a commercial 20S proteasome from human erythrocytes in the in-house prepared heavy-labeled sP20S or iP20S samples. The same set of 10 peptide sequences as the one optimized previously for the AQUA-based quantification of 20S proteasome and corresponding to four non-catalytic subunits and six catalytic subunits were followed by SRM (Supplementary Data 2). When the mixture of commercial and home-prepared sP20S were analyzed, all the peptides could be detected with high intensities, except the three peptides corresponding to the three catalytic immuno-subunits (Supplementary Figure 4A). This is in accordance with the expected composition of both light and heavy proteasomes analyzed. Indeed, human erythrocytes contain mainly the sP20S subtype (7, 8). Of note, the Light/Heavy ratios for the peptides corresponding to α3, α4, β6, and β7 non-catalytic subunits were very close (1.8 ± 0.1) and similar to the L/H ratios measured for the three peptides of the catalytic subunits β5, β1, and β2 (1.76 ± 0.03). This result shows that both commercial and home-prepared P20S proteasome samples contain the same proportion of 20S standard and immuno proteasome sub-types. Thus, as erythrocytes contain only sP20S, the in-house isotopically-labeled sP20S seems to exhibit a high purity. A purity of 102 ± 8 % was more precisely assessed by calculating the stoichiometry of the β5 subunit within total 20S

proteasome, using SRM and normalization with the light peptide signals from erythrocytes proteasome. Indeed, the β5 subunit is specific of sP20S and is never found in the other known 20S proteasome subtypes (except in some specific tissues like testes). When the in-house heavy-labeled iP20S was analyzed together with the commercial erythrocytes sP20S using the same SRM method, the heavy signals corresponding to β5i, β1i, and β2i, were very high compared to their light counterparts, as expected (Supplementary Figure 4B). The isotope-labeled iP20S contains 1.2 ± 0.1 % of sP20S; this corresponds to the measured stoichiometry of β5 within total 20S proteasome. This indicates that the isotope-labeled iP20S preparation can be considered as nearly pure. The two in-house heavy-labeled sP20S and iP20S preparations (that we will call "SILAC sP20S" and "SILAC iP20S" in the manuscript) were further used as internal standards to absolutely quantify total 20S proteasome but also to decipher the stoichiometry of 20S subtypes in different biological samples.

With these new tools in our hands, we used the same strategy as the one presented previously with the AQUA peptides to evaluate the SRM assay for total 20S quantification. Both SILAC purified proteasomes were used as internal standards; more precisely, they were added at an equimolar concentration (1260 ng/mL each) in the U937 cell protein lysate, previously depleted from endogeneous proteasome (using proteasome immuno-purification (2)) and spiked with increasing concentrations of commercial human erythrocyte proteasome (Supplementary Figure 5). The same four peptides sequences as the ones used for the AQUA first trial, LLDEVFFSEK, ALLEVVQSGGK, GAVYSFDPVGSYQR, and AIHSWLTR corresponding to α3, α4, β6, and β7 non-catalytic subunits, respectively, were analyzed as light and heavy (R($^{13}C_6$), and lysine, K($^{13}C_6$)) forms. Internal standard normalization with the heavy surrogates allowed absolute quantification of total 20S proteasome. Results show that, contrary to what was obtained with AQUA normalization strategy, internal standard normalization with the four heavy SILAC peptides correlates perfectly with the proteasome absolute quantification obtained by ELISA, both in terms of precision (max CV of 8.5 %), linearity (coefficients of determination above 0.99), and accuracy (mean global accuracy of 90 %) (Supplementary Figure 5). The tryptophan containing peptide AIHSWLTR gave results of a bit lower quality in terms of CVs and

accuracies, possibly because of differential oxidation of W. Thus, peptides containing M and W-oxidable amino acids were skipped in the final SRM assay. The advantage of using whole isotope-labeled proteasome complex as internal standard over the AQUA or even the PrEST approach (9) is that, at least theoretically, all proteotypic peptides of each subunit can be used for the quantification. The best peptides were however carefully optimized to reach a final SRM method including in total, 206 independent MS transitions (103 "light" transitions and 103 "heavy" transitions corresponding to heavy surrogate peptides) associated with optimized dwell times and voltages applied to the mass spectrometer (Supplementary Data 3).

The ELISA sandwich assay measures fully-assembled 20S proteasome only whereas the SRM results have been obtained independently from individual α and β non-catalytic subunits SRM-signals measured directly in human cell lysates. Thus, the adequation observed between the two independent assays suggests that the fraction of 20S subunits in the free form or in the course of assembly are very low in these cell lines and tissues. This is supported by the experimental observation that SRM signals of α and β non-catalytic subunits are hardly detectable in the low MW fractions of glycerol-gradient separated proteins from U937 cells and correlate with the ELISA quantification in the high MW fractions containing fully-assembled 20S proteasomes (Supplementary Figure 6A). Moreover, α and β non-catalytic subunits, which are sequentially incorporated during the 20S proteasome assembly process (10), are present in stoichiometric amounts both in the panels of eight cell lysates (Supplementary Figure 6B) and of 11 human tissues (Supplementary Figure 6C) which also argues that they are mainly incorporated into canonical fully-assembled proteasomes.

Altogether, these data prove the suitability of the new developed method for absolute quantification of 20S proteasome in human cell lysates and tissues.

2- Validation of the assay for determination of 20S subtypes stoichiometry and dynamics using a model of pro inflammatory cytokines-treated HeLa cells

An important goal of this work was, in addition to 20S proteasome absolute quantification, to determine the stoichiometry of the different proteasome subtypes in biological samples. Together with the detection of peptides corresponding to non-catalytic subunits, we constructed our method to also follow the six standard and immuno catalytic subunits of 20S proteasome. Three peptides and at least three transitions per peptide were optimized for each protein, except for β2 for which only two peptide sequences suited our selection criteria. This illustrates how optimization of peptide sequences is really an issue to meet the expected requirements for IDMS analysis of proteins (1). In this respect, methods relying on the spike-in of the whole heavy-labeled protein as internal standard, like PSAQ (11) or absolute SILAC (12, 13), seem superior to the PrEST methodology (9) where only a short sequence of each protein is used.

Internal normalization using equimolar spikes of our two home-made heavy sP20S and iP20S permitted to monitor precisely the dynamics of all 20S proteasome subunits in interferon γ-treated HeLa cells over a 96h kinetics. The ability of our method to accurately determine 20S proteasome subtypes stoichiometry was then assessed by considering the known stoichiometries of incorporation of catalytic subunits. Indeed, for each catalytic subunit type, the sum of the absolute quantities of the standard subunit and its immuno-counterpart should equal the amount of total proteasome. So, as detailed hereafter, there are four different methods to determine the absolute quantity of total 20S proteasome from our measurements and at each IFNγ-stimulation time:

1. total 20S proteasome = mean (α1-7, β3-4, β6-7): reference method (validated by ELISA)
2. total 20S proteasome = β1 + β1i
3. total 20S proteasome = β2 + β2i
4. total 20S proteasome = β5 + β5i (no β5t identified by LC MS/MS)

Importantly, all these data were obtained from the SRM analysis of 206 independent MS transitions; 103 light transitions were extracted and normalized with their 103 respective heavy counterparts

(Supplementary Data 3). Because it has been previously validated using ELISA, the 20S absolute quantity obtained from the SRM measurements of the non-catalytic subunits (method 1) served as a reference method. So, the accuracies of the SRM measurements of the 6 major catalytic subunits were assessed by comparing the 20S proteasome total quantities obtained from the signals of each catalytic subunit couple (β1/β1i, β2/β2i, and β5/β5i) (methods 2 to 4) with the one given by the reference method (method 1) (Supplementary Figure 7A). Our optimized SRM method permitted to achieve accuracies above 96% for all three catalytic subunits types. In comparison with SRM, accuracies measured using the TOP3 label-free (LF) MS quantification (metric calculated as the mean of the three highest peptides areas measured for each protein), and obtained from the same biological samples, were much lower (76% for β1/β1i, 43% for β2/β2i, 79% for β5/β5i) (Supplementary Figure 7A). The poor trueness obtained with β2/ β2i label-free quantification may be explained by the fact that one of the TOP3 tryptic peptide analyzed for β2 is detected in at least five different forms arising from missed cleavages. The amount of peptides containing missed cleavages sites has been reported to influence the absolute quantification (4). Global accuracies obtained with the SRM and label-free MS methods are 97 ± 2 % and 78 ± 12 %, respectively (β2/ β2i signals were excluded for the label-free MS method only) (Supplementary Figure 7B). Concerning method variability, both MS quantification methods give mean CVs below 15%, though SRM seems more reliable as no outliers (*ie* with CVs above 30%) were measured (Supplementary Figure 7C-D). These performances are in accordance with a previous report where we compared the ability of four different label-free MS approaches to determine 26S proteasome stoichiometry in various cell lines (14). So, these data confirm that label-free MS1-based quantification methods, which are straightforward and allow protein identification and quantification with the same data set, can only give a rough estimation of protein complexes stoichiometry (14, 15); these approaches are rather dedicated to the high throughput determination of changes in the relative abundances of protein complex subunits (16–18). Overall, these data indicate that our optimized SRM method is able to accurately and precisely report the absolute amounts of the six different 20S standard and immuno catalytic subunits.

II. Supplementary Figures Legend:

Supplementary Figure 1: Linearity, sensitivity, and precision of the LC-SRM method

A- Typical chromatogram obtained for the SRM analysis of ten selected peptides corresponding to four non-catalytic subunits (α3, α4, β6, and β7) and six catalytic subunits (β1, β2, β5, β1i, β2i and β5i). The signal corresponds to the sum of three transitions for each peptide. The equivalent of 5 fmoles of trypsin-digested commercial proteasomes purified from human erythrocytes was injected on column.

B- Calibration curves obtained for seven selected peptides after injection of increasing quantities of trypsin-digested commercial proteasomes purified from human erythrocytes. The equivalent of 0.5 to 500 fmoles of proteolyzed 20S proteasome were injected on column together with 70 fmoles of heavy AQUA surrogate peptides using the optimized method. The SRM method was based on the isotopic dilution of four heavy-labeled proteotypic peptides « AQUA peptides » (LLDEVFFSEK ($^{13}C_6$, $^{15}N_2$), ALLEVVQSGGK ($^{13}C_6$, $^{15}N_2$), GAVYSFDPVGSYQR ($^{13}C_6$, $^{15}N_4$), and AIHSWLTR ($^{13}C_6$, $^{15}N_4$)) from four different non catalytic subunits (α3, α4, β6, and β7, respectively) of 20S proteasome. The three transitions giving the highest intensities (from preliminary experiments) were analyzed for each peptide, as detailed in Supplementary Data 1. Light transitions signals were normalized with their respective heavy counterparts. The normalized transitions corresponding to a given peptide sequence were then averaged to give an intensity ratio Light/Heavy which is reported as a function of the quantity injected on column. Left panel: whole range of injected quantities. Right panel: zoom on the lowest quantities (0.5 to 5 fmoles). Three to five replicates were averaged and used for statistics.

C- Coefficients of variation on intensity ratios (Light/Heavy) measured in the two quantity ranges (25-500 fmol and 0.5-5 fmol). Three to five replicates were used for statistics.

Supplementary Figure 2: Validation of the LC-SRM quantification of total 20S proteasome in biological sample using AQUA peptides

A- Commercial 20S proteasome purified from Human erythrocytes was spiked at five different concentrations in a U937 cells human protein lysates previously depleted from endogeneous proteasome. Proteasome concentration was then measured by the ELISA reference method or by using the SRM approach. The SRM method was based on the isotopic dilution of four heavy-labeled proteotypic peptides « AQUA peptides » (LLDEVFFSEK ($^{13}C_6$, $^{15}N_2$), ALLEVVQSGGK ($^{13}C_6$, $^{15}N_2$), GAVYSFDPVGSYQR ($^{13}C_6$, $^{15}N_4$), and AIHSWLTR ($^{13}C_6$, $^{15}N_4$)) from four different non catalytic subunits (α3, α4, β6, and β7, respectively) of 20S proteasome. The three transitions giving the highest intensities (from preliminary experiments) were analyzed for each peptide, as detailed in Supplementary Data 1. Five microliters of an equivalent of 2.5 µg total proteins was injected on column. The peptides obtained after trypsin digestion were spiked with 70 fmol of the four AQUA peptides. Light transitions signals were normalized with their respective heavy counterparts. The normalized transitions corresponding to a given peptide sequence were then averaged. The signal from the heavy AQUA peptide was used to determine the quantity of the endogeneous peptide (light signal). The total proteasome concentration measured in the injected samples and given by each peptide sequence is presented. For each biological replicates, results from 3 replicates of injection were averaged. Three biological replicates were analyzed to obtain statistics.

B- and C- Coefficients of variation (B) and accuracies (D) were calculated for each peptide sequence. Coefficients of variation were calculated as ratios of the standard deviation over the mean of the values and expressed as a percentage. Accuracies were obtained as the ratio of the difference of the experimental value and the reference value over the reference value and expressed as a percentage. The reference value corresponds to 20S proteasome concentration obtained by the ELISA reference assay.

Supplementary Figure 3: workflow for heavy sP20S and iP20S production and quality assessment

Proteasome were affinity-purified from HEK EBNA cells, either untransfected (containing mainly the three catalytic subunits β5, β1 and β2 of sP20S) or transfected with the three immuno catalytic subunits β5i, β1i and β2i, so that these cells contain only iP20S, as shown using immuno-detection of these subunits. Cell cultures were performed in media containing the heavy forms of arginine, R($^{13}C_6$), and lysine, K($^{13}C_6$). Ten cellular doubling were performed in this medium to reach a 95% rate of heavy amino acids incorporation into protein (assessed by MS). Absolute quantities and purities of produced and purified heavy-labeled standard proteasome and immunoproteasome were then assessed using SRM thanks to the spike-in of a known quantity of commercial 20S standard proteasome from erythrocytes (as shown in supplementary Figure 4).

Supplementary Figure 4: **Absolute quantification and purity assessment of in-house produced isotope-labeled sP20S and iP20S**

LC-SRM analysis of 10 selected peptides of 20S proteasome in a mix of commercial sP20S from human erythrocytes and isotopically-labeled (R($^{13}C_6$) and K($^{13}C_6$) sP20S (A) and iP20S (B). These results were used to determine the absolute concentrations and purities of in-house prepared heavy-labeled sP20S (A) and iP20S (B), as follows. The light peptide signals from commercial sP20S from human erythrocytes were used as internal standards. An equivalent of 80 fmol of commercial sP20S was injected on column. Light and heavy signals of 3 transitions were analyzed for each peptide sequence corresponding to four 20S proteasome non-catalytic subunits (α3, α4, β6, and β7) and six 20S proteasome catalytic subunits (β1, β2, β5, β1i, β2i, and β5i), as detailed in Supplementary Data 2. For each biological replicate, results from 2 replicates of injection were averaged. Three biological replicates were analyzed to obtain statistics. Total 20S proteasome was calculated from the averaged signals given by the four non catalytic subunits (α3, α4, β6, and β7), by considering the initial quantity

of erythrocyte proteasome injected and the 95% rate of incorporation of arginine R($^{13}C_6$), and lysine K($^{13}C_6$) into sP20S and iP20S isotope-labeled proteasomes.

Supplementary Figure 5: validation of the strategy for absolute quantification of proteasome by LC-SRM using absolute SILAC

A- Correlation curve between the 20S proteasome concentrations measured by the ELISA reference method and those determined by SRM based on the signals given by different peptide sequences. Commercial 20S proteasome purified from Human erythrocytes was spiked at five different concentrations in a U937 cells human protein lysates previously depleted from endogeneous proteasome. Proteasome concentration was then measured by the ELISA reference method or by using the SRM approach. The SRM method was based on the isotopic dilution of in-house isotopicaly-labeled sP20S and iP20S (R($^{13}C_6$) and K($^{13}C_6$)) (Figure 1B). Four peptide sequences (LLDEVFFSEK, ALLEVVQSGGK, GAVYSFDPVGSYQR, and AIHSWLTR) from four different non-catalytic subunits (α3, α4, β6, and β7, respectively) of 20S proteasome were analyzed. The three transitions giving the highest intensities (from preliminary experiments) were analyzed for each peptide, as detailed in Supplementary Data 2. Endogeneous U937 proteins were spiked with increasing amounts of commercial proteasome (from human erythrocytes) and a fixed quantity of a mixture of isotopically-labeled sP20S and iP20S (1 pmol of each for 25µg total proteins). SRM analyses are detailed in the Experimental Procedures. For each biological replicate, results from 3 replicates of injection were averaged. Three biological replicates were analyzed to obtain statistics. The total proteasome concentration measured in the injected samples and given by each peptide sequence is presented. Details are available in the Experimental Procedures.

B- The total proteasome concentrations given by the SRM approach on the four peptide sequences were averaged to obtain a global correlation curve between the ELISA reference method and the SRM approach.

C-D Coefficients of variation (CV %) and accuracies (%) were calculated for each peptide sequence. Coefficients of variation were calculated as ratios of the standard deviation over the mean of the values and expressed as a percentage. Accuracies were obtained as the ratio of the difference of the experimental value and the reference value over the reference value and expressed as a percentage. The reference value corresponds to the 20S proteasome concentration obtained from the Elisa reference assay.

Supplementary Figure 6: SRM and ELISA analyses are consistent for the detection of proteasome subunits in the different glycerol gradient-separated fractions obtained from a cell lysate.

A- Analysis of glycerol-gradient separated proteins extracted from U937 cell lysates. Proteasome content was analyzed in the different fractions by the ELISA reference method or by using the LC-SRM approach. The SRM method was based on the analysis of three transitions of four proteotypic peptides (LLDEVFFSEK, ALLEVVQSGGK, GAVYSFDPVGSYQR, and AIHSWLTR) corresponding to four different non-catalytic subunits (α3, α4, β6, and β7, respectively) of 20S proteasome. Transitions were validated on the LC-MS signal thanks to the parallel monitoring of four heavy-labeled « AQUA peptides » (LLDEVFFSEK ($^{13}C_6$, $^{15}N_2$), ALLEVVQSGGK ($^{13}C_6$, $^{15}N_2$), GAVYSFDPVGSYQR ($^{13}C_6$, $^{15}N_4$), and AIHSWLTR ($^{13}C_6$, $^{15}N_4$)).

B- Quantities (as a percentage of the total 20S proteasome amount) of α and β non catalytic subunits measured in a panel of eight human cell lines. No significant difference is observed between the two distributions.

C- Quantities (as a percentage of the total 20S proteasome amount) of α and β non-catalytic subunits measured in a panel of 11 human tissues. No significant difference is observed between the two distributions.

Supplementary Figure 7: The LC-SRM method is able to precisely and accurately monitor 20S subtypes catalytic subunits

A- Method accuracy obtained from comparison of total 20S proteasome quantity obtained from non-catalytic subunits (reference values) and from β1/β1i, β2/β2i, and β5/β5i couples of catalytic subunits (experimental values). Accuracy were computed as follows: accuracy = (experimental value – reference value) / reference value * 100. Accuracies were calculated from quantifications obtained with the optimized SRM approach and with the TOP3 label-free quantification (LF) on the same biological samples. Twelve independent measurements were computed for each catalytic subunit type (3 biological replicates, 4 time points). The biological samples consist of HeLa cells stimulated for 0, 24, 72, or 96 h with IFNγ. The SRM method was based on the isotopic dilution of equimolar amounts of in-house isotopically-labeled sP20S and iP20S (R($^{13}C_6$) and K($^{13}C_6$)) in each total cell lysate obtained at each time point of cytokine stimulation. One peptide sequence (and three transitions for the light and the heavy surrogate) and three peptide sequences were analyzed for non-catalytic subunit (α1-7; β3,4,6,7) and catalytic subunits (β1,2,5,1i, 2i, 5i), respectively (see Supplementary Data 3 for more details on peptides and transitions). SRM data analyses are detailed in the experimental section. The label-free (LF) MS quantification method corresponds to the TOP3 metric where quantification values are calculated as the mean of the three highest peptides areas measured for each protein. Three biological replicates were analyzed to obtain statistics.

B- Global accuracies obtained from SRM and label-free (LF) absolute quantifications of catalytic subunits of 20S proteasome, as explained in A. Signals from the β2/ β2i couple were excluded for the label-free MS method only.

C- Coefficients of variation for each catalytic subunit on independent measurements obtained by label free (LF) and SRM quantification (n = 4).

D- Global variability (CV %) obtained from SRM and label-free (LF) absolute quantifications of catalytic subunits of 20S proteasome (n = 24).

Supplementary Figure 8: **The particular case of the α4 subunit within the human 20S proteasome**

A-B: FASTA sequences corresponding to ubiquitous α4 (O14818; PSMA7_Human) and to its testis-specific isoform called α4s (Q8TAA3; PSMA7L_HUMAN) and peptides chosen for their specific detection by SRM (A), and sequence alignment of the two proteins (B). The LC-SRM method could be applied to accurately quantify specifically the α4 protein (O14818) using 3 specific peptides (RPFGISALIVGFDFDGTPR, NYTDEAIETDDLTIK, and ILNPEEIEK).

C-D: Correlation between the absolute quantity of α4 (PSMA7 & PSMA7L) and total 20S proteasome (α4 excluded) in a panel of eight human cell lines (C) and in a panel of 11 protein lysates extracted from human tissues of broad origins (D). Quantifications were obtained using LC-SRM on peptides common to PSMA7 and PSMA7L isoforms. The LC-SRM method was based on the isotopic dilution of equimolar amounts of in-house isotopically-labeled sP20S and iP20S (R($^{13}C_6$) and K($^{13}C_6$)) in each cell line protein lysate. LC-SRM acquisition and data analyses are detailed in Experimental Procedures. Three biological replicates were analyzed.

Supplementary Figure 9: **Correlation between protein and mRNA for 20S proteasome subunits in human tissues**

A-D. Graph representing the protein intensities (from our MRM assay) against the mRNA counts (obtained from the human protein atlas (19)) measured from human tissues for the 20S proteasome subunits β5 **(A)**, β5i **(B)**, β1i **(C),** β2i **(D)**, α2 **(E)**, and α1 **(F)**. The resulting Pearson correlation coefficient and coefficient of determination are indicated on the graphs.

Supplementary Figure 10:

A- Stoichiometries of 20S proteasome subtypes in primary ADSCs derived from five different patients and cultivated during ten days at three O_2 levels (1%, 5%, and 20%). Calculations are detailed in Experimental Procedures.

B- Fold change in the stoichiometry of 20S proteasome-associated regulators amplified in hypoxia compared to normoxia. 20S proteasome complexes were immunopurified and the proportions of 20S-associated regulators were determined by label-free MS, as detailed previously (17, 20).

Supplementary Figure 11: **Effect of oxygen level on ASDCs proteins expressions**

A. Volcano plot representing the $\log_2$ ratio (hypoxia/normoxia) for each protein quantified and the corresponding p-value. The blue, red and grey dots represent the proteins more abundant in hypoxia (1% O_2), more abundant in normoxia, and not differentially expressed, respectively. Grey and green dashed lines represent the fold changes thresholds for proteins up- and down-regulated and the $p = 0.05$ threshold, respectively. B-C. Results from GO terms enrichment analysis for the proteins more abundant in normoxia (B) and more abundant in hypoxia (C). The number of proteins identified in each pathway (protein count) and the p-value ($-\log_{10}$ transformed) are represented on the graphs.

III. Supplementary Data List and Legends:

Supplementary Data n°1 : Peptides sequences, SRM transitions, and voltages applied for the LC-SRM analysis of 20S Proteasome using AQUA peptides (corresponding to Sup Fig. 1 & 2)

Supplementary Data n°2 : Peptides sequences, SRM transitions, and voltages applied for the LC-SRM analysis of 20S Proteasome using isotope-labeled whole proteasome complex (corresponding to Sup Fig. 4 & 5)

Supplementary Data n°3 : Peptides sequences, SRM transitions, and voltages applied for the LC-SRM analysis of 20S Proteasome using isotope-labeled whole proteasome complex (corresponding to Figures 2-5 and Sup Fig. 8 & 9)

Supplementary Data n°4 : Peptides sequences, SRM transitions, and voltages applied for Quality Controls (QC) (injection of 20 fmol of tryptic digest of betagalactosidase).

Supplementary Data n°5 : Experimental LOD and LLOQ obtained by injecting heavy-isotope labelled sP20S and iP20S spiked at increasing concentrations in a HeLa protein lysate.

Supplementary Data n°6 : Protein and Peptide identification data corresponding to Figure 3B.

Supplementary Data n°7: Protein (1st sheet) and Peptide (2nd sheet) identification data corresponding to Supplementary Figure 11. 3rd sheet: data used to plot supplementary Figure 11A and determine the over- or under-represented proteins. 4st sheet: varying proteins harboring the term "differentiation" in their GO Biological Processes.

Supplementary Data n°8 : Detailed description of mass spectrometry data sets deposited in repositories (raw and processed file names, sample type, biological replicate number, MS technical replicate number, analytical conditions).

IV. References:

1. Lange V, Picotti P, Domon B, Aebersold R (2008) Selected reaction monitoring for quantitative proteomics: A tutorial. *Mol Syst Biol* 4(1):222–235.
2. Bousquet-Dubouch MP, et al. (2008) Purification and proteomic analysis of 20S proteasomes from human cells. *Methods Mol Biol* 432:301–320.
3. Brun V, Masselon C, Garin J, Dupuis A (2009) Isotope dilution strategies for absolute quantitative proteomics. *J Proteomics* 72(5):740–749.
4. Schmidt C, Lenz C, Grote M, Lührmann R, Urlaub H (2010) Determination of protein stoichiometry within protein complexes using absolute quantification and multiple reaction monitoring. *Anal Chem* 82(7):2784–2796.
5. Shuford CM, et al. (2017) Absolute Protein Quantification by Mass Spectrometry: Not as Simple as Advertised. doi:10.1021/acs.analchem.7b00858.
6. Guillaume B, et al. (2010) Two abundant proteasome subtypes that uniquely process some antigens presented by HLA class I molecules. *Proc Natl Acad Sci U S A* 107(43):18599–604.
7. Claverol S, Burlet-Schiltz O, Girbal-Neuhauser E, Gairin JE, Monsarrat B (2002) Mapping and structural dissection of human 20 S proteasome using proteomic approaches. *Mol Cell Proteomics* 1(8):567–578.
8. Bousquet-Dubouch M-P, et al. (2009) Affinity Purification Strategy to Capture Human Endogenous Proteasome Complexes Diversity and to Identify Proteasome-interacting Proteins. *Mol Cell Proteomics* 8(5):1150–1164.
9. Zeiler M, Straube WL, Lundberg E, Uhlen M, Mann M (2012) A Protein Epitope Signature Tag (PrEST) library allows SILAC-based absolute quantification and multiplexed determination of protein copy numbers in cell lines. *Mol Cell Proteomics* 11(3):O111.009613.
10. Hirano Y, et al. (2006) Cooperation of Multiple Chaperones Required for the Assembly

of??Mammalian 20S Proteasomes. *Mol Cell* 24(6):977–984.

11. Kaiser SE, et al. (2011) Protein standard absolute quantification (PSAQ) method for the measurement of cellular ubiquitin pools. *Nat Methods* 8(8):691–6.

12. Hanke S, Besir H, Oesterhelt D, Mann M (2008) Absolute SILAC for accurate quantitation of proteins in complex mixtures down to the attomole level. *J Proteome Res* 7(3):1118–1130.

13. Geiger T, et al. (2011) Use of stable isotope labeling by amino acids in cell culture as a spike-in standard in quantitative proteomics. *Nat Protoc* 6(2):147–157.

14. Fabre B, et al. (2014) Comparison of label-free quantification methods for the determination of protein complexes subunits stoichiometry. *EuPA Open Proteomics* 4:82–86.

15. Wohlgemuth I, Lenz C, Urlaub H (2015) Studying macromolecular complex stoichiometries by peptide-based mass spectrometry. *Proteomics* 15(5–6):862–879.

16. Fabre B, et al. (2015) Deciphering preferential interactions within supramolecular protein complexes: the proteasome case. *Mol Syst Biol* 11(1):771.

17. Fabre B, et al. (2014) Label-free quantitative proteomics reveals the dynamics of proteasome complexes composition and stoichiometry in a wide range of human cell lines. *J Proteome Res* 13(6):3027–3037.

18. Oliviero G, et al. (2016) Dynamic Protein Interactions of the Polycomb Repressive Complex 2 during Differentiation of Pluripotent Cells. *Mol Cell Proteomics* 15(11):3450–3460.

19. Uhlen M, et al. (2015) Tissue-based map of the human proteome. *Science (80-)* 347(6220):1260419–1260419.

20. Fabre B, et al. (2013) Subcellular Distribution and Dynamics of Active Proteasome Complexes Unraveled by a Workflow Combining in Vivo Complex Cross-Linking and Quantitative Proteomics. *Mol Cell Proteomics* 12(3):687–699.
