# Supplementary material for: Mass Spectrometry-based Absolute Quantification of 20S Proteasome Status for Controlled Ex-vivo Expansion of Human Adipose-derived Mesenchymal Stromal/Stem Cells
Source: Mol Cell Proteomics. 2019 Jan 30;18(4):744–59. doi: 10.1074/mcp.RA118.000958 (PMC6442357; doi:10.1074/mcp.RA118.000958)

Sup Figure 1

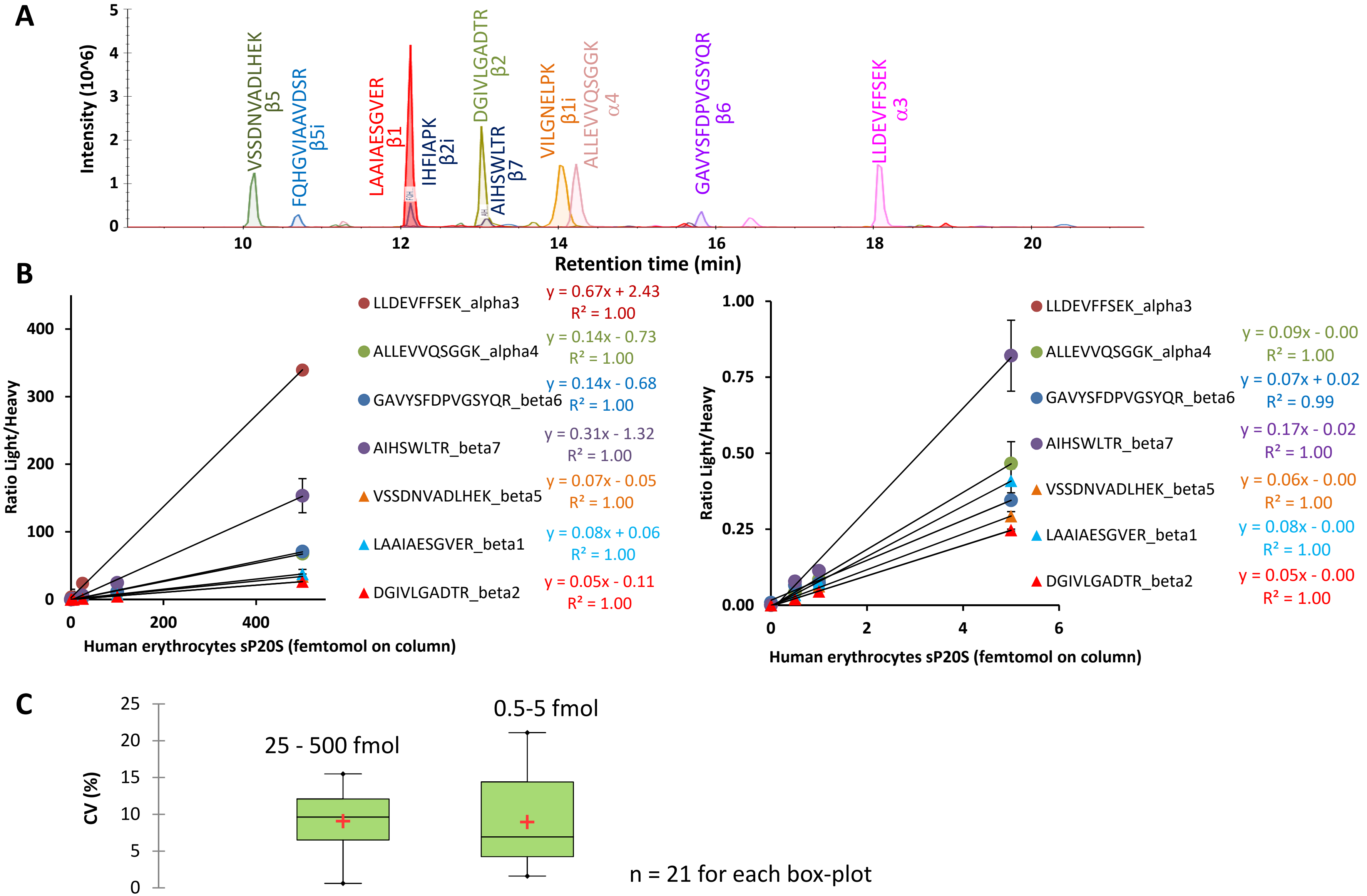

A
Intensity (10^6)
VSSDNVADLHEK β5
FQHGVIAAVDSR β5i
LAAIAESGVER β1
IHFIAPK β2i
DGIVLGADTR β2
AIHSWLTR β7
VILGNELPK β1i
ALLEVVQSGGK α4
GAVYSFDPVGSYQR β6
LLDEVFFSEK α3
Retention time (min)
B
Ratio Light/Heavy
Human erythrocytes sP20S (femtomol on column)
LLDEVFFSEK_alpha3
ALLEVVQSGGK_alpha4
GAVYSFDPVGSYQR_beta6
AIHSWLTR_beta7
VSSDNVADLHEK_beta5
LAAIAESGVER_beta1
DGIVLGADTR_beta2
y = 0.67x + 2.43 R² = 1.00
y = 0.14x - 0.73 R² = 1.00
y = 0.14x - 0.68 R² = 1.00
y = 0.31x - 1.32 R² = 1.00
y = 0.07x - 0.05 R² = 1.00
y = 0.08x + 0.06 R² = 1.00
y = 0.05x - 0.11 R² = 1.00
y = 0.09x - 0.00 R² = 1.00
y = 0.07x + 0.02 R² = 0.99
y = 0.17x - 0.02 R² = 1.00
y = 0.06x - 0.00 R² = 1.00
y = 0.08x - 0.00 R² = 1.00
y = 0.05x - 0.00 R² = 1.00
C
CV (%)
25 - 500 fmol
0.5-5 fmol
n = 21 for each box-plot

Sup Figure 2

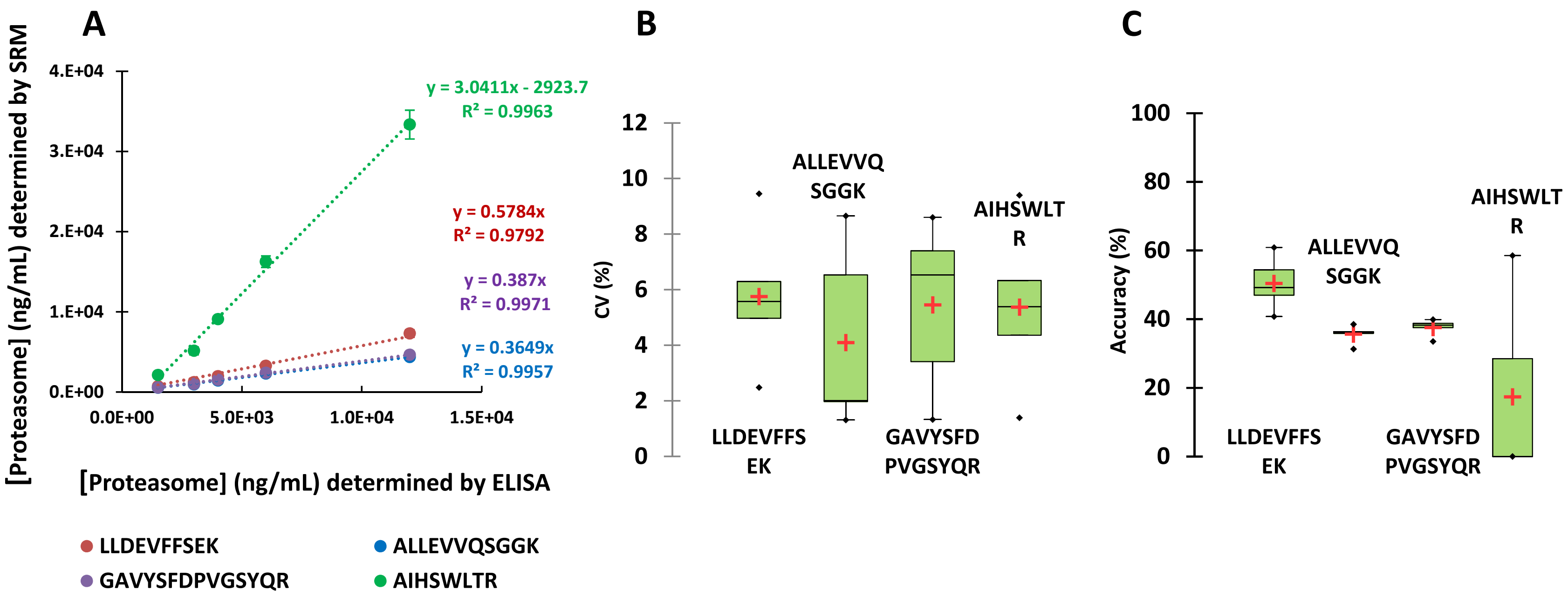
A
B
C
[Proteasome] (ng/mL) determined by SRM
[Proteasome] (ng/mL) determined by ELISA
4.E+04
3.E+04
2.E+04
1.E+04
0.E+00
0.0E+00
5.0E+03
1.0E+04
1.5E+04
y = 3.0411x - 2923.7
R² = 0.9963
y = 0.5784x
R² = 0.9792
y = 0.387x
R² = 0.9971
y = 0.3649x
R² = 0.9957
LLDEVFFSEK
ALLEVVQSGGK
GAVYSFDPVGSYQR
AIHSWLTR
CV (%)
12
10
8
6
4
2
0
ALLEVVQ SGGK
AIHSWLT R
LLDEVFFS EK
GAVYSFD PVGSYQR
Accuracy (%)
100
80
60
40
20
0

Sup Figure 3

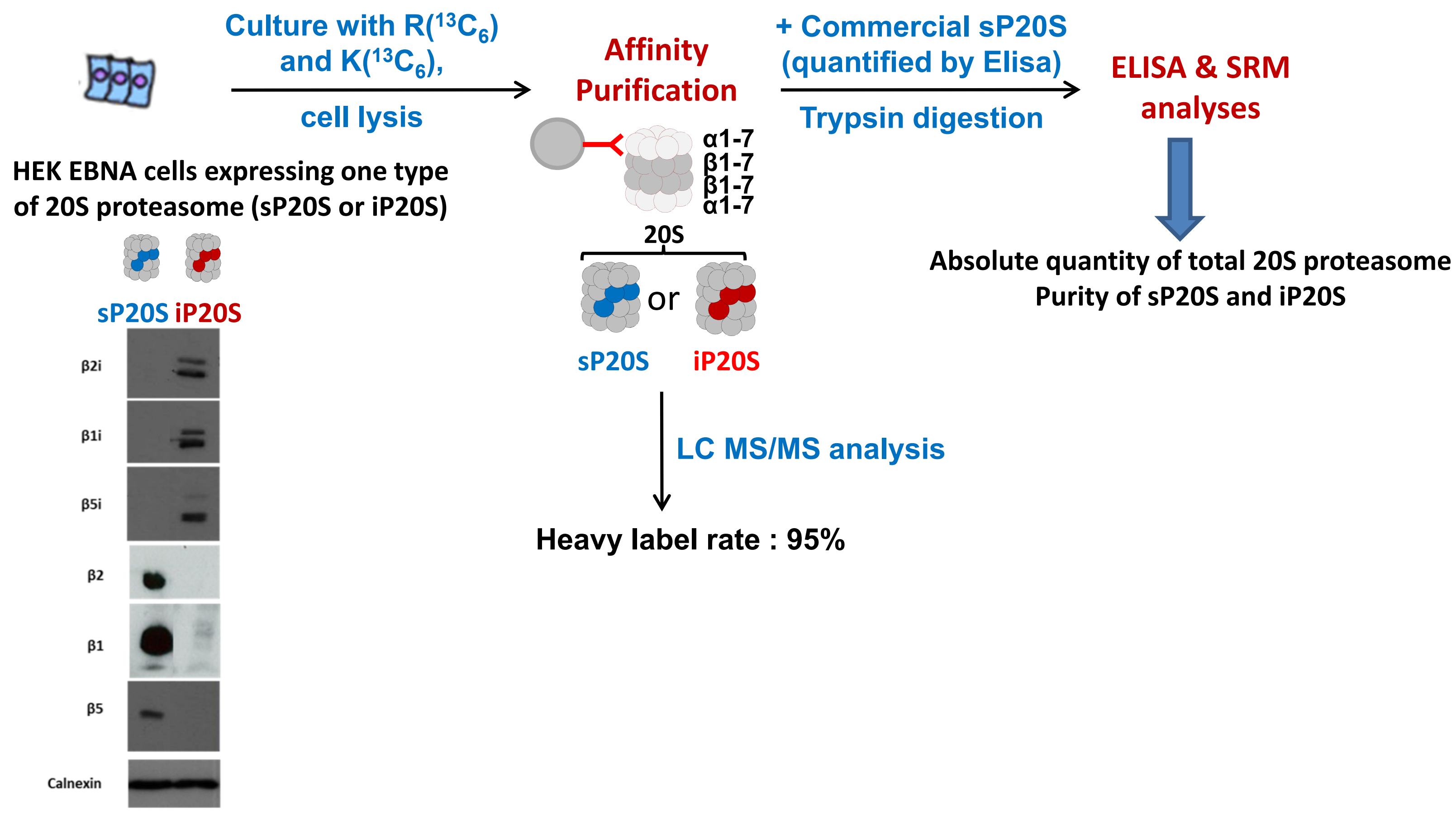

Culture with R($^{13}C_6$) and K($^{13}C_6$),
cell lysis
HEK EBNA cells expressing one type of 20S proteasome (sP20S or iP20S)
sP20S iP20S
β2i
β1i
β5i
β2
β1
β5
Calnexin
Affinity Purification
α1-7
β1-7
β1-7
α1-7
20S
or
sP20S
iP20S
LC MS/MS analysis
Heavy label rate : 95%
+ Commercial sP20S (quantified by Elisa)
Trypsin digestion
ELISA & SRM analyses
Absolute quantity of total 20S proteasome
Purity of sP20S and iP20S

Sup Figure 4

A

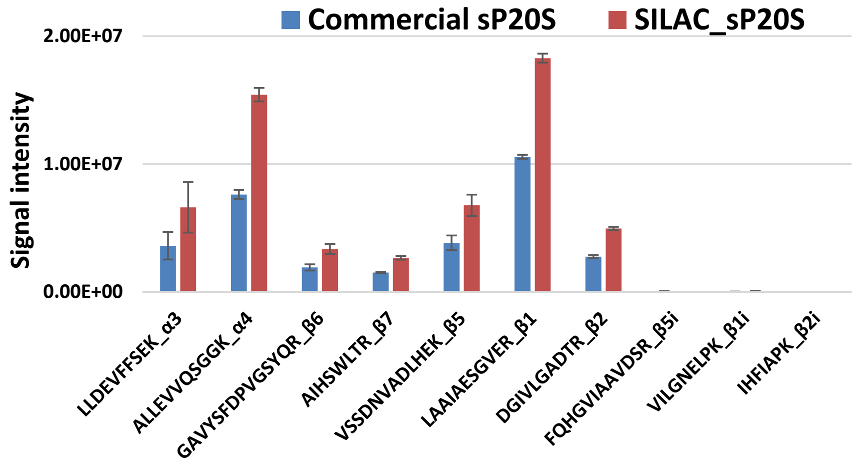

B

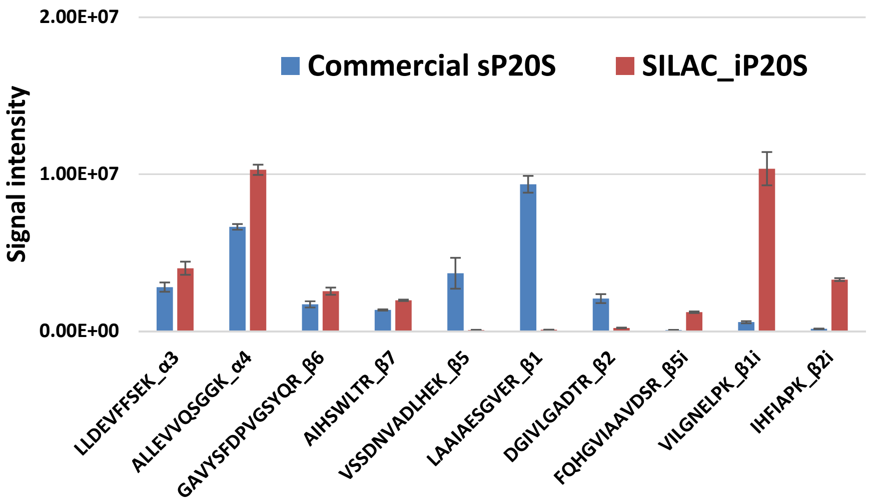

Sup Figure 5

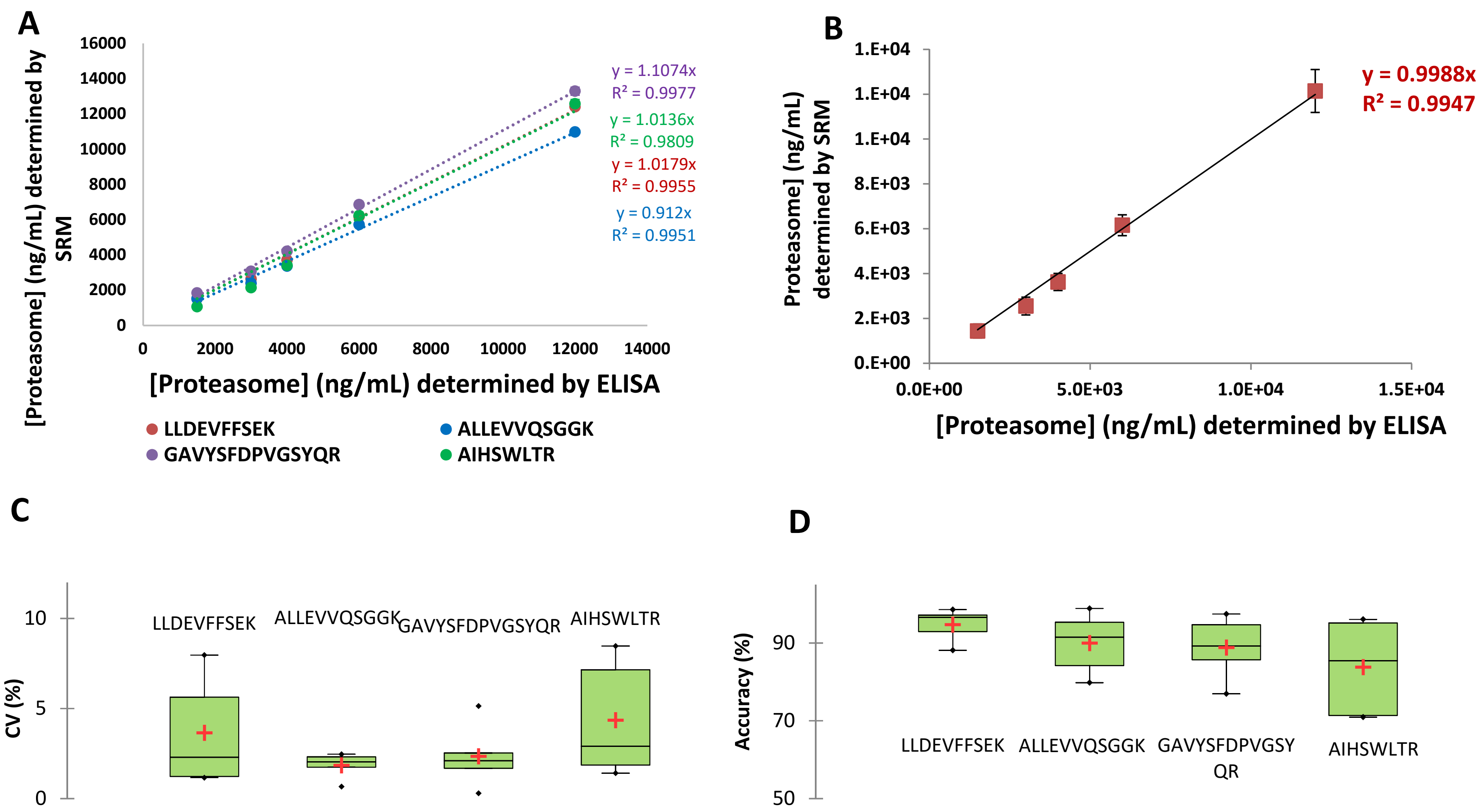
A
[Proteasome] (ng/mL) determined by SRM
16000
14000
12000
10000
8000
6000
4000
2000
0
2000
4000
6000
8000
10000
12000
14000
y = 1.1074x
R² = 0.9977
y = 1.0136x
R² = 0.9809
y = 1.0179x
R² = 0.9955
y = 0.912x
R² = 0.9951
[Proteasome] (ng/mL) determined by ELISA
LLDEVFFSEK
ALLEVVQSGGK
GAVYSFDPVGSYQR
AIHSWLTR
B
Proteasome] (ng/mL) determined by SRM
1.E+04
1.E+04
1.E+04
8.E+03
6.E+03
4.E+03
2.E+03
0.E+00
0.0E+00
5.0E+03
1.0E+04
1.5E+04
y = 0.9988x
R² = 0.9947
[Proteasome] (ng/mL) determined by ELISA
C
CV (%)
10
5
0
LLDEVFFSEK
ALLEVVQSGGK
GAVYSFDPVGSYQR
AIHSWLTR
D
Accuracy (%)
90
70
50
LLDEVFFSEK
ALLEVVQSGGK
GAVYSFDPVGSY QR
AIHSWLTR

Sup Figure 6

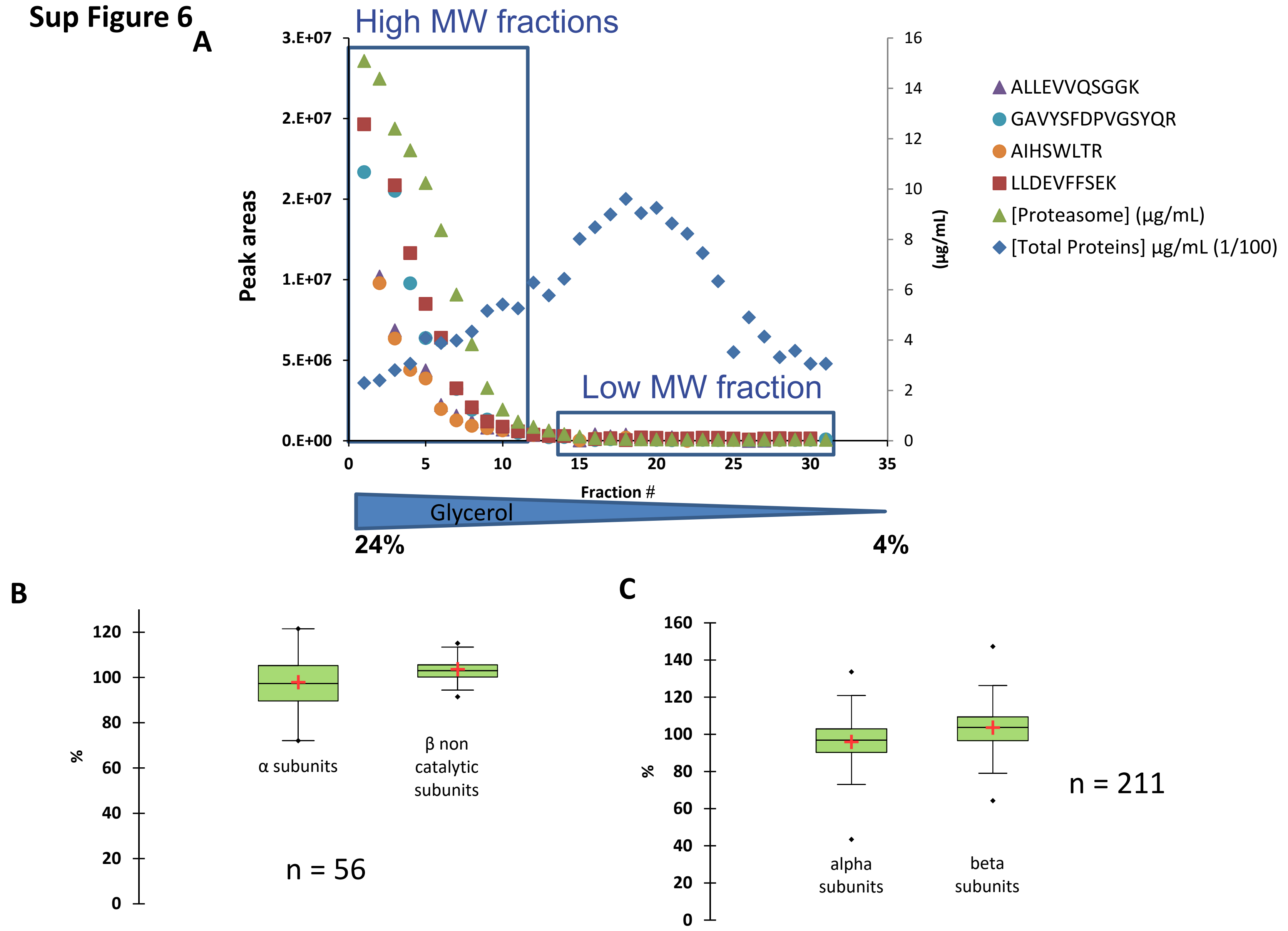
A
High MW fractions
Low MW fraction
Peak areas
(µg/mL)
Fraction #
Glycerol
24%
4%
ALLEVVQSGGK
GAVYSFDPVGSYQR
AIHSWLTR
LLDEVFFSEK
[Proteasome] (µg/mL)
[Total Proteins] µg/mL (1/100)
B
%
α subunits
β non catalytic subunits
n = 56
C
%
alpha subunits
beta subunits
n = 211

Sup Figure 7

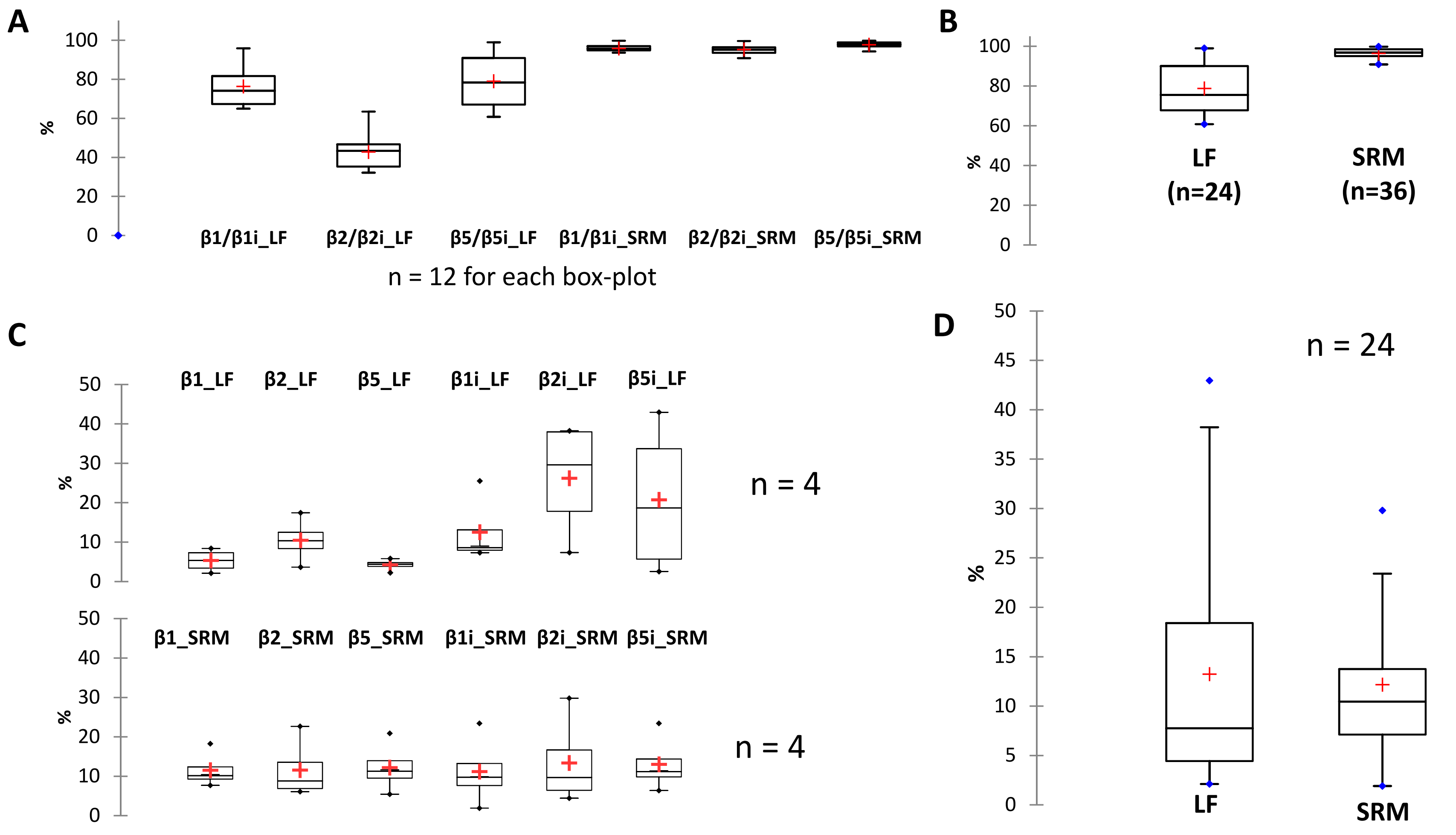
A
100
80
60
40
20
0
%
β1/β1i_LF
β2/β2i_LF
β5/β5i_LF
β1/β1i_SRM
β2/β2i_SRM
β5/β5i_SRM
n = 12 for each box-plot
B
100
80
60
40
20
0
%
LF (n=24)
SRM (n=36)
C
50
40
30
20
10
0
%
β1_LF
β2_LF
β5_LF
β1i_LF
β2i_LF
β5i_LF
n = 4
β1_SRM
β2_SRM
β5_SRM
β1i_SRM
β2i_SRM
β5i_SRM
n = 4
D
50
45
40
35
30
25
20
15
10
5
0
%
n = 24
LF
SRM

Sup Figure 8

A

Ubiquitous α4 : >sp|O14818|PSA7_HUMAN Proteasome subunit alpha type-7 OS=Homo sapiens GN=PSMA7 PE=1 SV=1
MSYDR**AITVFSPDGHLFQVEYAQEAVK**KGSTAVGVRGRDIVVLGVEKKSVAKLQDERTVR
KICALDDNVCMAFAGLTADARIVINRARVECQSHR**LTVEDPVTVEYITR**YIASLKQRYTQSN
GR<u>**RPFGISALIVGFDFDGTPR**</u>LYQTDPSGTYHAWKANAIGRGAKSVREFLEK<u>**NYTDEAIET**</u>
<u>**DDLTIK**</u>LVIK**ALLEVVQSGGK**NIELAVMRRDQSLK<u>**ILNPEEIEK**</u>YVAEIEKEKEENEKKKQKKAS

Testis-specific α4 called α4s : >sp|Q8TAA3|PSA7L_HUMAN Proteasome subunit alpha type-7-like OS=Homo sapiens GN=PSMA8 PE=2 SV=3
MASRYDR**AITVFSPDGHLFQVEYAQEAVK**KGSTAVGIRGTNIVVLGVEKKSVAKLQDERT
VRKICALDDHVCMAFAVLTIFIGLTADARVVINRARVECQSHK**LTVEDPVTVEYITR**FIATLK
QKYTQSNGRRPFGISALIVGFDDDGISRLYQTDPSGTYHAWKANAIGRSAKTVREFLEKNY
TEDAIASDSEAIKLAIK**ALLEVVQSGGK**NIELAIIRRNQPLKMFSAKEVELYVTEIEKEKEEAEK
KKSKKSV

Peptides common to α4 and α4s ➔ total α4 position subunit quantity
<u>**Peptides specific to α4 (PSMA7)**</u>➔ used to deduce α4s absolute quantity

B

```
O14818 PSA7_HUMAN      1   --MSYDRAITVFSPDGHLFQVEYAQEAVKKGSTAVGVRGRDIVVLGVEKKSVAKLQDERT   58
Q8TAA3 PSMA8_HUMAN     1   MASRYDRAITVFSPDGHLFQVEYAQEAVKKGSTAVGIRGTNIVVLGVEKKSVAKLQDERT   60
                               ********************************:** :*******************

O14818 PSA7_HUMAN     59   VRKICALDDNVCMAFA------GLTADARIVINRARVECQSHRLTVEDPVTVEYITRYIA   112
Q8TAA3 PSMA8_HUMAN    61   VRKICALDDHVCMAFAVLTIFIGLTADARVVINRARVECQSHKLTVEDPVTVEYITRFIA   120
                           *********:******      *******:************:**************:**

O14818 PSA7_HUMAN    113   SLKQRYTQSNGRRPFGISALIVGFDFDGTPRLYQTDPSGTYHAWKANAIGRGAKSVREFL   172
Q8TAA3 PSMA8_HUMAN   121   TLKQKYTQSNGRRPFGISALIVGFDDDGISRLYQTDPSGTYHAWKANAIGRSAKTVREFL   180
                           :***:******************** **  *********************.**:*****

O14818 PSA7_HUMAN    173   EKNYTDEAIETDDLTIKLVIKALLEVVQSGGKNIELAVMRRDQSLKILNPEEIEKYVAEI   232
Q8TAA3 PSMA8_HUMAN   181   EKNYTEDAIASDSEAIKLAIKALLEVVQSGGKNIELAIIRRNQPLKMFSAKEVELYVTEI   240
                           *****::** :*. :***.******************::**:* **::. :*:* **:**

O14818 PSA7_HUMAN    233   EKEKEENEKKKQKKAS                                             248
Q8TAA3 PSMA8_HUMAN   241   EKEKEEAEKKKSKKSV                                             256
                           ****** ****.**:
```

C

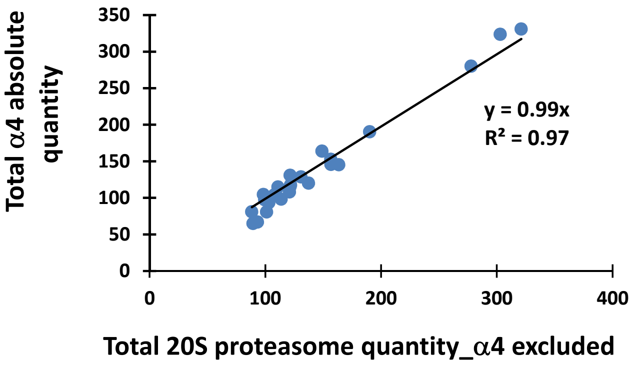

D

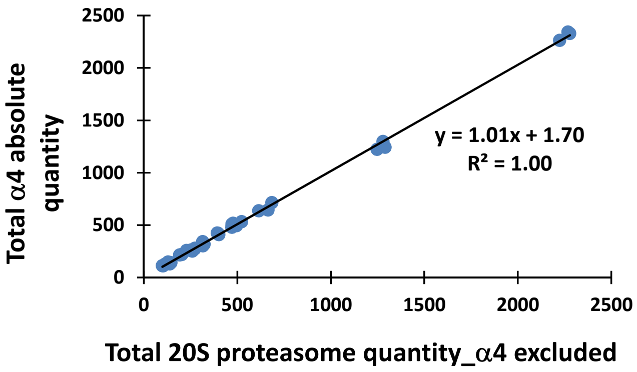

Sup Figure 9

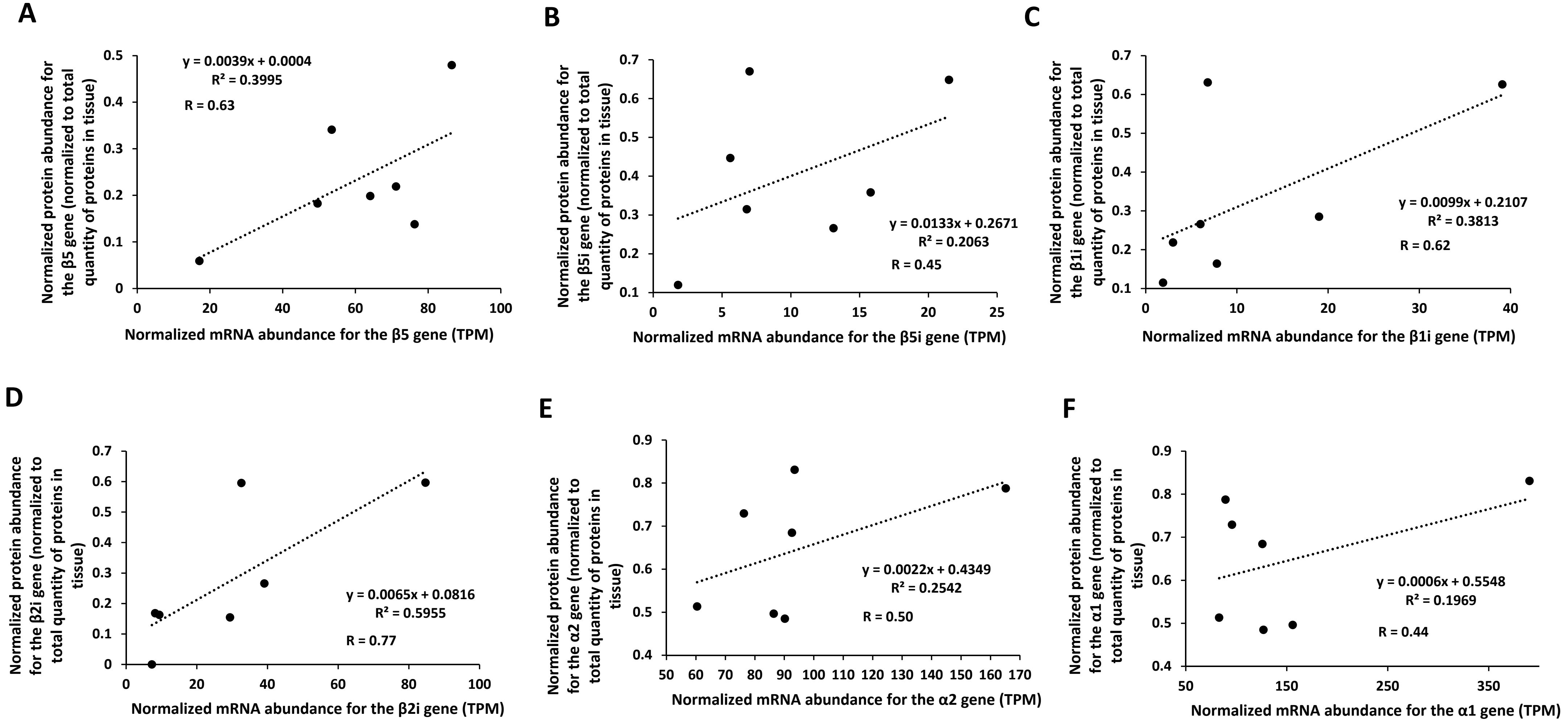
A
y = 0.0039x + 0.0004
R² = 0.3995
R = 0.63
Normalized protein abundance for the β5 gene (normalized to total quantity of proteins in tissue)
Normalized mRNA abundance for the β5 gene (TPM)
B
y = 0.0133x + 0.2671
R² = 0.2063
R = 0.45
Normalized protein abundance for the β5i gene (normalized to total quantity of proteins in tissue)
Normalized mRNA abundance for the β5i gene (TPM)
C
y = 0.0099x + 0.2107
R² = 0.3813
R = 0.62
Normalized protein abundance for the β1i gene (normalized to total quantity of proteins in tissue)
Normalized mRNA abundance for the β1i gene (TPM)
D
y = 0.0065x + 0.0816
R² = 0.5955
R = 0.77
Normalized protein abundance for the β2i gene (normalized to total quantity of proteins in tissue)
Normalized mRNA abundance for the β2i gene (TPM)
E
y = 0.0022x + 0.4349
R² = 0.2542
R = 0.50
Normalized protein abundance for the α2 gene (normalized to total quantity of proteins in tissue)
Normalized mRNA abundance for the α2 gene (TPM)
F
y = 0.0006x + 0.5548
R² = 0.1969
R = 0.44
Normalized protein abundance for the α1 gene (normalized to total quantity of proteins in tissue)
Normalized mRNA abundance for the α1 gene (TPM)

Sup Figure 10

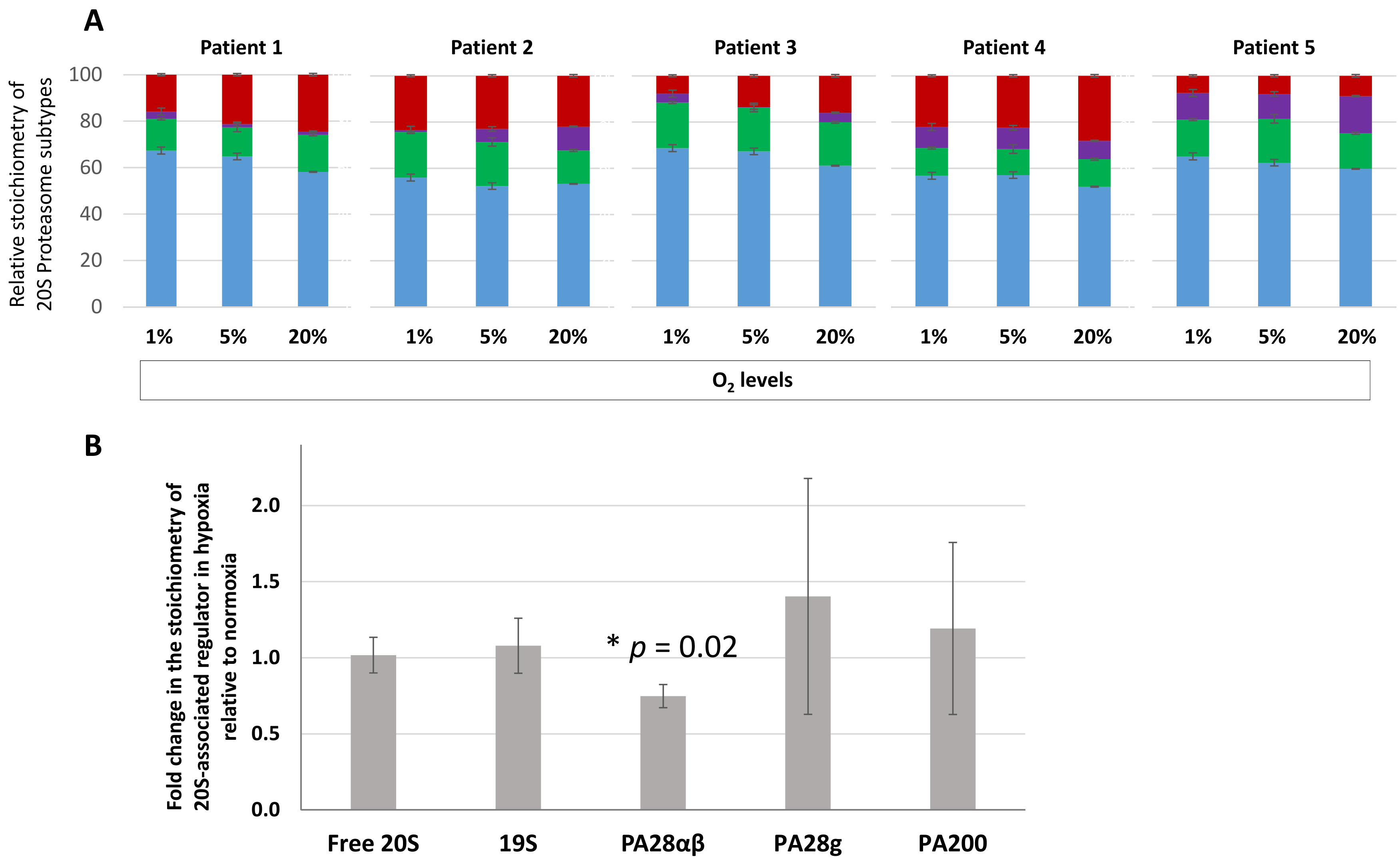

Sup Figure 11

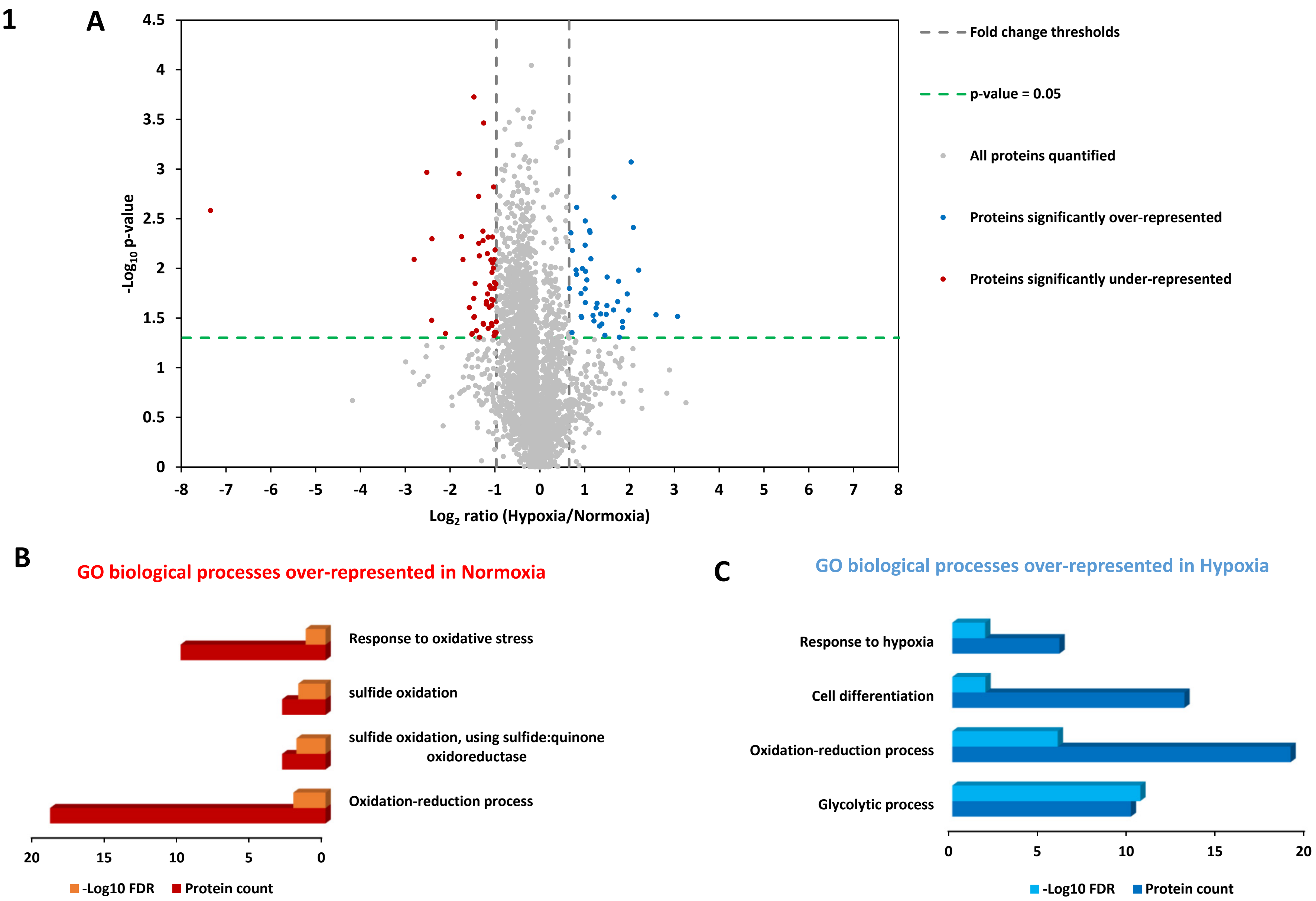

Supplement: Supplemnatry Figures [file 139223_1_supp_270975_plj9gm.pdf]
